# Molecular mechanisms of colorectal cancer: from carcinogenesis and molecular classification to targeted therapy

**DOI:** 10.1186/s10020-026-01635-w

**Published:** 2026-11-04

**Authors:** Shorouk Aldeyarbi, Ehab Elhanafy, Maha Saber-Ayad, Ali Hussein Abu Almaaty, Alaa Sagheer, Mohamed Kamel Hassan

**Affiliations:** 1 Biotechnology Program, Faculty of Science, Port Said University, Port Said, Egypt; 2 Faculty of Medicine, Port Said University, Port Said, Egypt; 3 College of Medicine, University of Sharjah, Sharjah, UAE; 4 School of Medicine, New Giza University, Giza, Egypt; 5 Department of Computer Science, College of Computer Sciences and Information Technology, King Faisal University, Al-Ahsa, Saudi Arabia

**Keywords:** CRC, Genetic alterations, Tumor microenvironment, Chromosomal instability, Microsatellite instability, CpG island methylator phenotype, DNA methylation, Microbiome, Immune regulation, Machine learning, Digital pathology, Molecular signaling pathways, EGFR signaling, Wnt/β-catenin signaling, TP53; TGF-β signaling, Targeted therapy, Immunotherapy, Precision medicine

## Abstract

Colorectal cancer (CRC) is a heterogeneous disease that results from the progressive accumulation and interactions at multiple levels of genetic alterations, which lead to the transformation of normal colonic epithelium into malignant colorectal tumors. Understanding the molecular basis of CRC pathogenesis, together with the interactions between genetic alterations, epigenetic modifications, environmental and lifestyle factors, is essential for improving prevention, diagnosis, prognosis, and treatment strategies. This review aims to provide a critical reference point for understanding the temporal dynamics of colorectal carcinogenesis, the evolution of molecular classification systems, including their computational and machine-learning foundations and emerging image-based prediction approaches, molecular drivers and the tumor microenvironment (TME) of CRC by summarizing the most investigated molecular pathways involved in CRC pathogenesis and their role as biomarkers for early CRC diagnosis based on the three main molecular mechanisms of colorectal carcinogenesis: chromosomal instability (CIN), microsatellite instability (MSI), and CpG island methylator phenotype (CIMP). Importantly, emerging evidence reveals that the TME acts as an important factor in driving CRC progression by regulating the immune system through dynamic cellular and molecular interactions. In particular, tumor metabolic reprogramming and microbiota-driven alterations act as major drivers of therapy resistance and immune escape. Combining these mechanisms will enhance biomarker discovery and aid in developing precision medicine for patients with CRC. This review also highlights the regulatory roles of key signaling pathways, including Wnt/β-catenin, epidermal growth factor receptor (EGFR), tumor protein p53 (TP53), and transforming growth factor-beta (TGF-β) signaling, during CRC initiation and progression, while further addressing current targeted therapies and emerging precision-based and experimental therapeutic strategies.

## Introduction

**Fig. 1:**
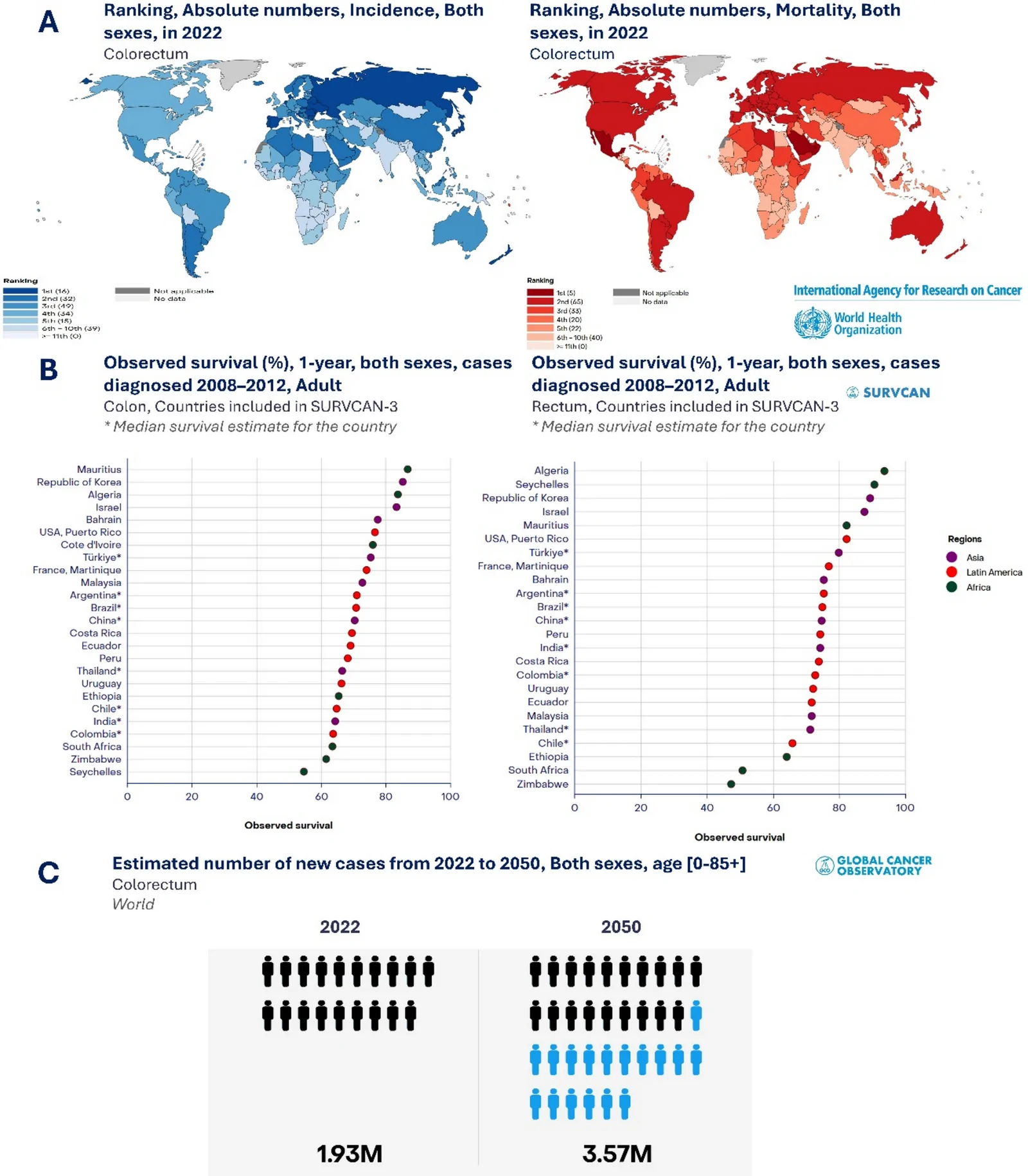
Global burden and survival estimate of CRC. **A** Global ranking of CRC incidence and mortality in 2022 for both sexes across countries. **B** One-year observed survival (%) for colon and rectal cancer among adults diagnosed between 2008–2012 in selected countries included in SURVCAN-3. **C** Estimated global number of new colorectal cancer cases, projected from 2022 to 2050. The legends indicate the number of countries within each ranking category. Sources: GLOBOCAN 2022, SURVCAN-3, and Global Cancer Observatory

**Fig. 2:**
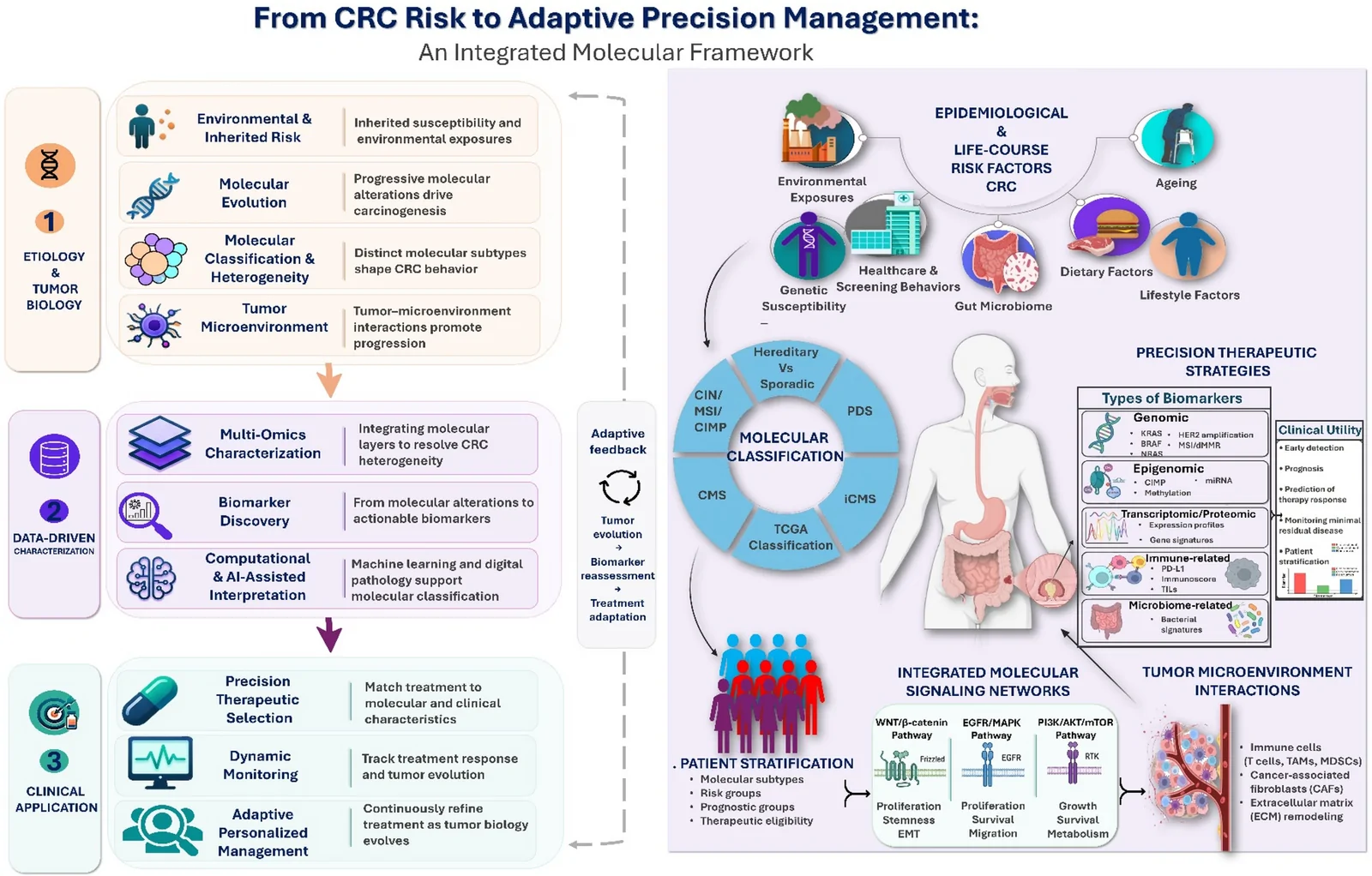
Integrated framework of CRC development and precision oncology. **A** The framework shows how studying the etiology and risk factors of CRC, together with molecular characterization based on integrating data from different aspects of the disease, can support clinical decisions and help select precise therapies. Molecular tools assist in monitoring treatment response and adjusting disease management. Finally, the data collected throughout this journey provide valuable feedback to inform future assessment and management. **B** The diagram shows how epidemiological risk factors, molecular classification systems, and biomarker profiles contribute to patient stratification and clinical management. Aberrant activation of major signaling pathways interacts with the tumor microenvironment to promote tumor progression, immune evasion, and therapeutic resistance. These interconnected molecular and microenvironmental mechanisms collectively influence treatment response and support precision medicine approaches in CRC (some graphical elements were created using BioRender). Abbreviations: CIN, chromosomal instability; MSI, microsatellite instability; CIMP, CpG island methylator phenotype; CMS, consensus molecular subtypes; PDS, pathway-derived subtypes; Wnt, Wingless/Integrated signaling pathway; EGFR, epidermal growth factor receptor; MAPK, mitogen-activated protein kinase; RAS, rat sarcoma viral oncogene; RAF, rapidly accelerated fibrosarcoma; MEK, MAPK/ERK kinase; ERK, extracellular signal-regulated kinase; PI3K, phosphoinositide 3-kinase; AKT, protein kinase B; mTOR, mammalian target of rapamycin; RTK, receptor tyrosine kinase; KRAS, Kirsten rat sarcoma viral oncogene; BRAF, v-Raf murine sarcoma viral oncogene homolog B1; NRAS, neuroblastoma RAS viral oncogene homolog; HER2, human epidermal growth factor receptor 2; dMMR, deficient mismatch repair; miRNA, microRNA; PD-L1, programmed death-ligand 1; ctDNA, circulating tumor DNA; CTCs, circulating tumor cells; TAMs, tumor-associated macrophages; MDSCs, myeloid-derived suppressor cells; CAFs, cancer-associated fibroblasts; ECM, extracellular matrix; EMT, epithelial–mesenchymal transition

CRC arises from the epithelial lining of the colon and rectum. The proximal and distal colon are the most common anatomical sites of CRC, each exhibiting distinct molecular characteristics that influence tumorigenesis (Yang et al. 2024). CRC typically develops over many years, with its incidence increases with age. While incidence is decreasing among older adults in many countries with high socio-demographic index that implement screening programs, early-onset CRC (< 50 years) continues to rise worldwide (Sung et al. 2025). Tumorigenesis begins with normal colonic epithelial cells progressing to adenomatous polyps, which subsequently evolve into invasive adenocarcinoma. This stepwise process is the adenoma-carcinoma sequence (Pino and Chung 2010). CRC is categorized as one of the primary malignancies due to its high mortality and morbidity rate (Fakhri 2017). In 2022, CRC occupied the third rank among predominant malignancies worldwide, in which form 1,926,118 new cases, accounting for 9.6% of total cancer diagnoses (Fig. 1A). CRC is the second leading cause of cancer-related death globally, accounting for 903,859 deaths (9.3%) in 2022 according to Global Cancer Observatory (GLOBOCAN) estimates (Bray et al. 2024). According to GLOBOCAN 2022, globally, the proportion of new CRC cases was highest in Europe (12.0%), followed by Asia (9.8%), Latin America and the Caribbean (9.4%), North America (6.9%), and Africa (5.9%). The highest CRC fatalities occurred in Europe at about 12.5%, then 9.8% in Latin America and the Caribbean, 9.4% in North America, 8.5% in Asia, and 6.0% in Africa. In addition to the global incidence and mortality distribution, Fig. 1B demonstrates country-specific differences in one-year survival rates for colon and rectal cancer, while Fig. 1C illustrates the expected increase in global CRC cases by 2050. Collectively, these results illustrate the differences not only in the prevalence of CRCs in various parts of the world, but also in terms of the survival outcomes and future prevalence of this disease. In particular, the disparity seen in the survival rates of these patients can be attributed to differences in screening practices, early diagnosis, and access to effective treatment and healthcare services among different regions. Intra-heterogeneity is one of the crucial factors in CRC heterogeneity characteristics, influencing both tumor behavior and treatment outcomes. This complexity arises from various factors, including genetic diversity, microenvironmental influences, pathobiological parameters, molecular characteristics, and gene expression patterns (Lee 2017). There are many ways to classify CRC, CIN, MSI, and CIMP are the three primary mechanisms. Several publications have previously covered different aspects of CRC. In our review, we focus on deciphering the dynamicity and heterogeneity of the disease multi-omics and molecular pathways, which inform the concept of precision medicine and the fact that no single therapy can fit all patients. We also argue whether the currently used classifications properly serve prognostication and the treatment approaches. We first discuss the temporal dynamics of colorectal carcinogenesis, followed by an overview of the evolution of molecular classification in CRC and the major molecular pathways underlying its development. Then we briefly describe the CRC tumor microenvironment, demonstrate clinically relevant molecular biomarkers, and discuss current as well as emerging therapeutic strategies. Finally, we highlight the major challenges and future perspectives in CRC research and clinical management, with an emphasis on advancing precision medicine and improving personalized patient care (Fig. 2A and B).

### *Temporal dynamics of colorectal carcinogenesis and longitudinal molecular epidemiology*

CRC is a complex gastrointestinal disorder that poses significant challenges to researchers and clinicians. Many experts attribute CRC risk to factors such as heredity, environmental and lifestyle factors. Although inherited susceptibility contributes to CRC risk, most cases arise through a complex interplay between genetic predisposition and environmental exposures (Wan et al. 2020). The epidemiology varies substantially across countries at different stages of socioeconomic development. In high-income nations, widespread screening programs and advances in treatment have contributed to declining incidence and mortality rates despite a persistently high disease burden. In contrast, many low- and middle-income countries continue to face limited access to screening and healthcare resources, resulting in delayed diagnosis and poorer outcomes. Moreover, economic development is often accompanied by lifestyle transitions characterized by increased consumption of energy-dense diets, excess body weight, sedentary behavior, unhealthy dietary habits, consumption of processed meats, tobacco, and alcohol intake. Conversely, higher consumption of dietary fiber, calcium-rich foods, dairy products, and regular aspirin use have been associated with a reduced risk of CRC. Emerging evidence also suggests that probiotics, such as those found in yogurt, may exert protective effects (Giovannucci 2022; Keum and Giovannucci 2019).

Consequently, the prevalence, timing, duration, and cumulative effects of environmental and lifestyle risk factors differ considerably among populations, potentially influencing the molecular pathways involved in colorectal carcinogenesis. Furthermore, differences in population age structure and cancer registry quality may contribute to variations in reported incidence rates. Collectively, these observations suggest that CRC develops through heterogeneous pathogenic trajectories shaped by life-course exposures and contextual factors, which may ultimately contribute to differences in tumor biology, clinical presentation, and patient outcomes across populations (Keum and Giovannucci 2019).

The CRC develops gradually in multistep processes that are characterized by the progressive accumulation of genetic alterations, histological, and morphological changes over time. Colorectal tumorigenesis occurs in several sequential phases, including initiation, promotion, progression, and metastasis. Accordingly, normal colon cells gradually transform into invasive carcinoma, through the sequential accumulation of mutations in *APC*, *KRAS*/*NRAS*, *PIK3CA*, *BRAF*, and *TP53*, together with alterations in their associated signaling pathways. These genetic alterations drive the transition from dysplasia and adenoma to carcinoma and ultimately invasive CRC. When metastatic spread occurs, the liver represents the most frequent site of dissemination, followed by the lungs and bones (Keum and Giovannucci 2019; Simon 2016; ALTamimi et al. 2026).

Early-onset CRC (EOCRC) risk factors are best identified through prospective longitudinal studies rather than cross-sectional, small-scale cohort analyses, or case–control studies, which may lack important covariates and sufficient statistical power (Laskar et al. 2025). The epidemiology of CRC is shifting towards a birth cohort effect, which provides additional insight into CRC etiology, risk stratification, and the biological mechanisms underlying current disease patterns among populations and groups who have shared similar events and exposures, such as diet, physical inactivity, antibiotic use, and environmental exposures that occurred early in life and accumulated over decades. For instance, the birth cohort effect in CRC has been observed across multiple successive generations, particularly among individuals born in 1960 and later, including *Generation X* (1965–1980) and *Millennials* (1981–1996), who exhibit rising EOCRC incidence and unique epidemiological features (Gupta et al. 2024). However, the traditional CRC screening programs were designed mainly for older adults because CRC was considered a disease of aging, but more cases occur in younger people; existing screening strategies may miss individuals who are at risk. Therefore, innovative screening approaches are needed to detect CRC earlier in younger populations (Li et al. 2024; Papila et al. 2026).

To bridge the gap between prospective epidemiology, which examines exposures over decades, and tumor molecular pathology, which investigates the biological changes occurring in CRCCRC, medical research has shifted to a new research design, the prospective cohort incident-tumor biobank method (PCIBM). A study may include the follow-up of more than 50,000 cancer-free participants by periodically administering questionnaires to collect information on family and personal history of diseases (including cancers), environmental exposures, and lifestyle factors. In addition, semiquantitative food frequency questionnaires (FFQs) can be used to assess dietary intake. These large-scale prospective cohort studies allow the evaluation of long-term risk factors before and after tumor diagnosis, while tumor tissue from participants who subsequently develop incident CRC is biobanked and molecularly analyzed. For instance, three U.S. prospective cohort studies used in the PCIBM framework are the Nurses'''''''' Health Study (NHS), which enrolled 121,701 female registered nurses in 1976; the Health Professionals Follow-up Study (HPFS), which enrolled 51,529 male health professionals in 1986; and the Nurses'''''''' Health Study II (NHS II), which enrolled 116,430 female registered nurses in 1989 (Ogino et al. 2026).

Herein, a recent study used the HPFS (NCT00005182) follow-up study to assess the relationship between age and tumor vascular features by conducting a multivariate analysis integrating a wide range of molecular pathological, and clinical data. According to the results, EOCRC patients tended to have fewer tumor blood vessels (both total CD34⁺ vessels and CD34⁺LAMB1⁺ vessels) in the TME than CRCs diagnosed in older patients. In addition, the authors suggested that, if future studies confirm this observation, reduced tumor vascularity could serve as a histological biomarker of EOCRC and provide new insights into the distinct biology and causes of CRC arising in younger adults (Matsuda et al. 2026).

Furthermore, the NHS and HPFS cohorts have also been used to demonstrate the association between the use of nonsteroidal anti-inflammatory drugs (NSAIDs), such as aspirin, and overall survival among patients with nonmetastatic CRC (stage I–III). The study was conducted based on previous demonstrations that colorectal neoplasia development can be reduced by aspirin intake. Aspirin inhibits cyclooxygenase 2 (COX-2), which catalyzes the production of prostaglandins and related eicosanoids from arachidonic acid, thereby limiting inflammation and tumor cell proliferation (Chan et al. 2007). According to the study results, after a median follow-up of 11.8 years, regular aspirin use after CRC diagnosis was associated with lower CRC-specific mortality (15% vs. 19%; hazard ratio (HR) = 0.71, 95% confidence interval (CI): 0.53–0.95) and overall mortality (35% vs. 39%; HR = 0.79, 95% CI: 0.65–0.97) compared with nonuse. The benefit was particularly evident among patients with COX-2-overexpressing tumors (HR = 0.39, 95% CI: 0.20–0.76) (Chan et al. 2009).

Moreover, the NHS and HPFS cohorts were used to investigate the association between a Western-style diet and CRC incidence. A Western-style diet, characterized by high consumption of high-fat foods, refined grains, processed meat, and red meat, with lower consumption of whole grains, vegetables, and fruits, may be associated with a higher abundance of *pks*⁺ *Escherichia coli* (*E. coli*) in the intestine. These bacteria harbor the 54-kb polyketide synthase (*pks*) pathogenicity island, which encodes enzymes responsible for colibactin biosynthesis. Colibactin has a carcinogenic role and may contribute to colorectal carcinogenesis by alkylating DNA, inducing DNA double-strand breaks, causing DNA mutations, and promoting genomic instability. The study found that a Western-style diet was strongly associated with the development of CRC containing high levels of colibactin-producing *pks*⁺ *E. coli* (HR = 3.45), but not with tumors containing low or undetectable levels of *pks*⁺ *E. coli*, suggesting that interactions between diet and the gut microbiota may contribute to colorectal carcinogenesis (Arima et al. 2022).

Collectively, the processes of CRC carcinogenesis can be viewed as dynamic, temporally changing phenomena rather than a stable disease state. Using longitudinal methods to integrate environmental risk factor exposures, molecular changes, and clinical outcomes would yield more information about the mechanisms driving colorectal carcinogenesis and its heterogeneity.

Building on the temporal dynamics of CRC carcinogenesis, advances in molecular profiling have led to the development of classification systems that better define CRC heterogeneity. The following section discusses the evolution of molecular classification in CRC and its implications for understanding tumor biology.

### Evolution of molecular classification in CRC

CRC heterogeneity arises from the accumulation of distinct molecular alterations during tumor initiation and progression, resulting in tumors with different biological behaviors, clinical outcomes, and therapeutic responses. Consequently, researchers have continually sought to better understand its underlying biology, resulting in the development of subsequent classification systems designed to capture different aspects of CRC heterogeneity (Dunne and Arends 2024; Dang et al. 2024; Saoudi González et al. 2023).

In the 1990 s, CRC was traditionally classified into hereditary and sporadic forms based on molecular events and environmental factors underlying tumor development (Fearon and Vogelstein 1990; Poulogiannis 2010; Brewer et al. 1993). Around 2010, research identified three principal molecular pathways driving CRC carcinogenesis: CIN pathway, MSI pathway, and CIMP pathway, which provided a molecular framework for understanding CRC carcinogenesis (Pino and Chung 2010; Worthley and Leggett 2010; Søreide et al. 2009). This framework was further refined by The Cancer Genome Atlas (TCGA) project in 2012, which integrated multi-omics data and confirmed a largely DNA-based classification of CRC (Dunne and Arends 2024; Muzny et al. 2012; Parmar 2022). Subsequently, transcriptomic profiling led to the development of the consensus molecular subtypes (CMSs) classification in 2015, which are MSI immune subtype (CMS1), canonical subtype (CMS2), metabolic subtype (CMS3), and mesenchymal subtype (CMS4). Moreover, CRC patients exhibiting CIN and microsatellite stability (MSS) are distributed among the consensus molecular subtypes CMS2, CMS3, and CMS4 (Guinney et al. 2015). In 2022, single-cell RNA sequencing (scRNA-seq) was used to elucidate gene expression, gene regulatory networks, and DNA copy number variation (CNV) in CRC epithelial cells and to reclassify CRC into a single-cell intrinsic consensus molecular subtype (iCMS) (Joanito et al. 2022). More recently, the Pathway-Derived Subtypes (PDS) based classification was proposed in 2024. Utilizing biological activation state information and gene ontology to further characterize CRC heterogeneity (Malla et al. 2024). A summary of the major CRC classification systems is presented in Table 1.

**Table 1:** Evolution of molecular classification systems in CRC

Classification system	Year	Basis of classification	Subtypes/Groups	Key molecular features	REF
Hereditary vs. Sporadic CRC	1990s	Molecular events and environmental factors underlying tumor development	Hereditary CRC; Sporadic CRC	Hereditary CRC includes HNPCC (Lynch syndrome) and FAP, both caused by inherited germline mutations; Sporadic CRC lacks a familial predisposition and typically arises from chromosomal instability and somatic mutations in key driver genes	Fearon and Vogelstein 1990; 2010; Brewer et al. 1993)
CIN/MSI/CIMP classification	Around 2010	Three principal molecular pathways driving CRC carcinogenesis	CIN; MSI; CIMP (serrated pathway)	CIN pathway, MSI pathway, and CIMP pathway	Pino and Chung 2010; Worthley and Leggett 2010; Søreide et al. 2009)
TCGA classification	2012	Multi-omics molecular information	CIN CRC (~ 84%); MSI CRC (~ 13–16%)	Epigenetic, genetic, and transcriptional patterns; DNA-based classification; MSI group exhibits DNA methylation that induces gene repression of many tumor suppressor genes	Dunne and Arends 2024; Muzny et al. 2012; 2022)
Consensus Molecular Subtypes (CMS)	2015	Bulk RNA expression profiling	CMS1 (MSI immune); CMS2 (canonical); CMS3 (metabolic); CMS4 (mesenchymal)	CMS1 includes CIMP-H, *BRAF* mutation, MSI-H, and low CIN hypermutated phenotype; CMS2 has activated WNT and MYC marks with strong epithelial signature; CMS3 is frequently associated with *KRAS* mutations and metabolic pathways deregulation; CMS4 is depicted by activated TGF-β signaling, complement-mediated inflammatory system activation, and extracellular matrix remodeling	Guinney et al. 2015)
Single-cell intrinsic Consensus Molecular Subtypes (iCMS)	2022	Single-cell RNA sequencing (scRNA-seq)	iCMS2; iCMS3	iCMS2 is predominantly composed of MSS tumors, including CMS2 tumors (96%) and 50% of fibrotic CMS4 tumors; iCMS3 includes the majority of MSI-H tumors and a subgroup of MSS tumors, including 97% of CMS1 tumors, 98% of CMS3 tumors, and half of CMS4 tumors with fibrosis	Joanito et al. 2022)
Pathway-Derived Subtypes (PDS)	2024	Biological activation state information and gene ontology	PDS1; PDS2; PDS3	PDS1 tumors are highly proliferative with good prognosis; PDS2 tumors are associated with stroma, immune-rich and intermediate prognosis; PDS3 tumors are transcriptionally inert and slow cycling tumors exhibiting neural-like transcriptional features with a poor prognosis	Malla et al. 2024)

These CRC classification systems have important clinical implications because CRC is characterized by its spatial heterogeneity within the same patient and interpatient heterogeneity (between different patients). The intratumoral heterogeneity of this cancer, a single tumor, is composed of multiple genetically and epigenetically distinct populations of cancer cells known as clones. Clones originate from a common ancestral cancer cell but acquire additional mutations and molecular changes as the tumor evolves. Accordingly, each clone within the same tumor possesses unique characteristics, such as growth rate, metastatic potential, and the ability to evade immune surveillance. Some clones may respond effectively to treatment, while others may survive due to resistant genetic features and subsequently drive disease progression or relapse (Saoudi González et al. 2023; Schmitt and Greten 2021). Thus, understanding clonal heterogeneity is essential for explaining variations in treatment response, disease progression, and prognosis in patients with CRC. For instance, using gene expression data from a subset of 443 colon cancer samples, Marisa et al. identified six molecular subtypes with distinct characteristics (Marisa et al. 2013). By distinguishing these subtypes, the classification guides the identification of distinct molecular markers unique to each subtype (Dang et al. 2024).

Therefore, the transition from a "One gene, one drug" strategy to a "Multi-gene, multi-molecule, multi-drug" strategy is necessary in CRC, as treatment decisions that rely on a single predictive biomarker are often insufficient to capture the complexity of tumor biology and resistance mechanisms (Dang et al. 2024). For instance, many studies show that tumors with wild-type *KRAS* and *NRAS* are sensitive and responsive to anti-EGFR monoclonal antibody treatments such as cetuximab and panitumumab. Nevertheless, many patients with wild-type *KRAS* do not respond to anti-EGFR therapy. They develop resistance to therapy. Research suggests that mutations in *BRAF, PDGFRA, ERBB2, FGFR1,* and *MEK1* can activate alternative signaling pathways. This allows the tumor to continue growing even when EGFR is blocked and highlights the drawbacks of single-site targeted therapy (Dang et al. 2024; Misale et al. 2014; Zimmer et al. 2014). Patients with CMS2 have high expression of EGFR ligands and activation of epithelial growth signaling pathways. This makes them sensitive to cetuximab. However, some CMS2 tumors develop resistance to EGFR mAbs through copy-number amplification of *ERBB2* and insulin-like growth factor 2 (*IGF2*). These changes activate alternative growth pathways that bypass EGFR inhibition. Thus, a combination therapy of insulin-like growth factor 1 receptor (IGF1R) inhibitors and pan-ERBB can improve treatment efficacy when compared with single-agent approaches (Dienstmann et al. 2017). Another study using a cellular model showed that combining IGF1R inhibition with autophagy suppressors can impede CRC metastasis (Mahgoub et al. 2026).

Overall, these classification systems serve as a platform for patient stratification by identifying distinct molecular and biological characteristics associated with different prognoses, treatment responses, and outcomes. Building on this, these systems highlight the need to understand the major molecular pathways underlying CRC heterogeneity.

#### Computational and machine learning foundations of CRC molecular classification

It is worth noting that each of the classification systems summarized in Table 1 is the product of a distinct computational strategy applied to molecular data, from consensus clustering and random-forest classification of bulk transcriptomes to single-cell clustering and pathway-level class discovery. These methods, and their emerging counterparts in image-based prediction, are discussed below.

Unlike the earlier classification systems, which were primarily based on clinicopathological and molecular characteristics, the more recent CRC molecular classification systems were derived through computational analyses of large-scale molecular datasets and supported by advances in machine learning. The CMS classification was established by integrating six transcriptomic classification systems using network-based consensus clustering, in which subtype associations were quantified using Jaccard similarity and hypergeometric testing, followed by Markov Cluster Algorithm (MCL)-based consensus clustering to define the four CMS groups. A Random Forest classifier was then used to assign new samples to the four CMS groups (Guinney et al. 2015). The classifier and the subtype calls it produces are, in practice, inseparable. In other words, the fraction of samples the classifier leaves unclassified, its handling of mixed tumors, and its sensitivity to expression platform have themselves become a subject of study (Trinh et al. 2017).

Subsequently, iCMS classification was generated from scRNA-seq data using computational quality-control pipelines, graph-based clustering, principal component analysis, differential gene expression analysis, and pseudo-bulk transcriptomic profiling to resolve epithelial cell heterogeneity (Joanito et al. 2022). As with CMS, a dedicated single-sample classifier has since been developed so that the iCMS call can be made on one tumor at a time without requiring a full cohort for normalization (Tsantoulis et al. 2025). More recently, the PDS classification integrated ssGSEA-derived pathway activity scores with computational clustering to define biologically distinct CRC subtypes, and subsequently employed a deep learning-based ResNet model to predict PDS directly from hematoxylin and eosin (H&E)-stained whole-slide images (WSIs) (Malla et al. 2024). Read together, CMS, iCMS and PDS trace a trajectory in which the molecular biology of CRC and the computational methods used to partition it have advanced in step.

More recently, advances in Artificial Intelligence (AI) have further extended computational approaches in CRC by enabling deep learning (DL) models trained on routine H&E-stained WSIs to predict clinically relevant molecular features, including MSI/dMMR. Kather et al. first showed that a deep residual network could predict MSI status directly from H&E histology in gastrointestinal cancers, without any additional stain or assay (Kather et al. 2019). This finding was subsequently hardened into a clinical-grade, multi-site validation by Echle et al., who reported that deep learning could flag MSI and mismatch-repair deficiency on routine slides faster and more cheaply than molecular testing (Echle et al. 2020), and a diagnostic study by Yamashita et al. found that such a model could match, and in their cohort exceed, the MSI calls of experienced gastrointestinal pathologists on whole-slide images (Yamashita et al. 2021). A systematic review and, more recently, a quantitative meta-analysis have since consolidated this evidence base and set out the conditions, principally large and diverse training cohorts, under which the approach is reliable (Echle et al. 2021; Li et al. 2025). This approach offers a more cost-effective and accessible strategy for pre-screening patients and may complement conventional immunohistochemistry (IHC)- and polymerase chain reaction (PCR)-based molecular testing.

Furthermore, the development of image-based consensus molecular subtype (imCMS) represents a transition from transcriptomic-based classification toward image-based molecular prediction, where DL models can infer CMS groups directly from H&E-stained WSIs (Sirinukunwattana et al. 2021). By linking tissue morphology with molecular classification, this approach provides a potentially simpler, faster, and more accessible alternative to conventional gene expression profiling while preserving the ability to characterize CRC heterogeneity. DL-based histology analysis has also been extended beyond MSI/dMMR prediction to identify additional clinically relevant molecular alterations. Since POLE-mutated MSS tumors share hypermutated profiles and overlapping histopathological features with MSI tumors, DL models trained on H&E-stained WSIs have demonstrated the potential to simultaneously detect MSI and POLE alterations from routine pathology images (Gustav et al. 2024). This approach may facilitate more comprehensive molecular screening and identify patients who could benefit from immunotherapy but may otherwise remain undetected by conventional testing strategies. These are early-stage tools, and their limitations, batch effects between scanners, the need for external validation, and reduced performance in under-represented populations, are well documented and should temper any claim of clinical readiness.

### Core molecular pathways driving chromosomal instability in CRC

The CIN subtype is one of the major genomic instability in CRC that drives cancer development (Geigl et al. 2008). CIN results from (1) large-scale changes in chromosome numbers, including aneuploidy or polyploidy, and (2) the loss of heterozygosity (LOH) caused by chromosomal structural alterations, including deletions, duplications, translocations, and complex chromosomal rearrangements (Pino and Chung 2010). The imbalances in gene dosage caused by CIN affect oncogenes and tumor suppressor genes, some of which function as driver genes. Also, the effect extends to the microRNAs (miRNAs) and results in aberrant gene expression that leads to the progression from adenoma to carcinoma. CIN tumors are associated with karyotypic abnormalities. The most notable abnormalities causing mutations in oncogene pathways or tumor suppressor gene pathways were chromosome alterations in 1q, 8q, 13q, and 20q through gain of extra segments in those arm regions, while chromosomal segment loss significantly involved CRC in chromosomes 1p, 8p, 17p, and 18q arm regions (Sylvester and Vakiani 2015; Hassan et al. 2014; Wang et al. 2016). In addition, the accumulation of mutations in specific oncogenes identifies chromosomal instability tumors, in which these mutations lead to subsequent tumorigenic pathways, such as KRAS (and less frequently BRAF). Tumor suppressor gene mutations lead to APC (Wnt signaling pathway) or p53 pathway (Tsang et al. 2014; Markowitz et al. 2009).

#### Wnt/β-catenin signaling pathway

Within the Wnt Signaling Pathway, the canonical Wnt/β-catenin-dependent pathway is an intracellular signal transduction cascade triggered by Wnt proteins. Wnt proteins are signaling molecules that regulate numerous cellular processes, including organogenesis, motility, polarity, stem cell renewal, and cell fate. They are a large protein family whose name originates from the Drosophila Wingless and mouse Int-1 (Patapoutian and Reichardt 2000; Brown 1998). Upregulation or downregulation of Wnt signaling has catastrophic consequences, and defective Wnt signaling plays a role in several human pathologies, e.g., skeletal abnormalities, breast cancer, and colon cancer (Logan and Nusse 2004; Komiya and Habas 2008). In CRC, Wnt/β-catenin signaling pathway abnormal activation occurs via several factors: (1) Germline mutations in the adenomatous polyposis coli (*APC)* suppressor gene are located on chromosome 5q22.2 and are responsible for about 60%–80% of FAP cases. FAP is a hereditary condition marked by hundreds or thousands of adenomas/polyps in the colon or rectum, which develop during adolescence and young adulthood. (2) *APC* somatic allelic deletion is considered a chromosomal instability subtype and is described in sporadic CRC. (3) *APC* gene epigenetic inactivation through promoter hypermethylation (identified in 18% of primary colorectal carcinoma and adenoma cases). (4) mutations in genes such as *AXIN2* (which produces the scaffolding Axin protein) and the *CTNNB1* gene (which produces the β-catenin protein) (Esteller 2000; Coppedè et al. 2014; Stanczak et al. 2011).

In a healthy cellular state, the transcriptional coactivator β-catenin is regulated by the Wnt signaling. When Wnt target genes are not required, the absence of Wnt stimulation suppresses their expression. Under these conditions, a protein complex known as the β-catenin destruction complex, consisting of the APC protein, casein kinase 1 (CK1), glycogen synthase kinase 3 (GSK-3), Axin (scaffolding protein), and E3-ubiquitin ligase b-TrCP, promotes the degradation of cytoplasmic β-catenin. However, β-catenin accumulates in the cytoplasm and subsequently translocates to the nucleus, where it binds to TCF/Lef proteins and activates the transcription of Wnt target genes. This occurs when Wnt proteins bind to cell surface receptors and prevent β-catenin degradation. For normal healthy cellular development, precise activation and inhibition of Wnt signaling target genes are required (Behrens et al. 1979; Stamos and Weis 2013).

The *APC* suppressor gene mutation arises in the CIN pathway from the earliest stage of CRC. The cellular abnormality comes from the lack of APC protein, a main component of the β-catenin destruction complex, which leads to Wnt signaling pathway activation and accumulation of cytoplasmic β-catenin that subsequently will translocate and bind to TCF/Lef proteins in the nucleus, thus promoting cancerous cell proliferation, migration, and invasion (Macdonald 2009; Yan 2024; Nguyen 2018).

##### KRAS mutations and signaling

*The Ras superfamily*. The Ras superfamily consists of small guanosine triphosphatases (GTPases), which are monomeric GTP-binding proteins that function as molecular switches in eukaryotic cells. In humans, the Ras superfamily comprises more than 150 members (Wennerberg et al. 2005). The most crucial member of the RAS superfamily implicated in CRC is *KRAS*. Approximately 30–50% of CRC cases harbor activating mutations in *KRAS* (Santini et al. 2008). *KRAS* mutation, like the *APC* mutation, which represents early genetic events leading to the CIN pathway, promoting adenoma-to-carcinoma progression (Takayama et al. 2001).

##### KRAS mutations and clinical implications

The *KRAS* gene is on chromosome 12, which encodes a 21 kDa protein; therefore, p21protein is an alternate name for the protein (Meng et al. 2021). The *KRAS* gene has six exons, while mutations in this gene occur primarilyin three exons: exons 2 (codons 12 and 13), 3 (codons 59 and 61), and 4 (codons 117 and 146) (Schwartzberg et al. 2014; Loupakis et al. 2009; Douillard et al. 2013; Hajdúch et al. 2010; Carta et al. 2006). Mutations in exon 2 are reported in more than 50% of CRC patients, and 90% of mutations of exon 2 are point substitutions (2024; Ferreira et al. 2022). Moreover, these cases are more susceptible to poor prognosis and drug therapy resistance (Pilozzi et al. 2021; Amado et al. 2023). Codon 12 mutations have a stronger oncogenic phenotype than codon 13 mutations (Table 2). The mutation frequency is approximately 23% for codon 13, followed by codon 61 (~ 2%) (Meng et al. 2021).

**Table 2:** Major *KRAS* mutations in CRC and their pathological association

*KRAS* Mutation	Codon	Amino Acid Change	Frequency in CRC (%)	Pathological Association	Ref
*KRAS*G12D	12 Exon (2)	Gly → Asp	13%	Highest proportion of GTP-bound KRAS, resulting in enhanced KRAS activation; associated with tumor progression and poor prognosis	2021; Hofmann et al. 2022)
*KRAS*G12V	12 Exon (2)	Gly → Val	9%	Associated with increased autophagy, enhanced cell proliferation, aggressive tumor behavior, and poor prognosis	Ferreira et al. 2022; Hofmann et al. 2022)
*KRAS*G12C	12 Exon (2)	Gly → Cys	~ 3%	Associated with worse prognosis in metastatic CRC; clinically relevant as a target for *KRAS* G12C inhibitors	Jones et al. 2017; Nassar et al. 2021)
*KRAS*G13D	13 Exon (2)	Gly → Asp	7%	Associated with increased risk of recurrence, particularly anastomotic recurrence, and poor prognosis	Hofmann et al. 2022; Jones et al. 2017)

Alternative splicing of exon 4 A and exon 4B of the *KRAS* gene produces two mRNA variants (*KRAS* 4 A and *KRAS*4B) (Chen et al. 2000). Abubaker et al., reported that the expression of the *KRAS* 4 A isoform was predominantly localized in the cytoplasm, and its overexpression was associated with left-sided colon adenocarcinoma. On the other hand, the *KRAS*4B protein isoform was in the nucleus, and its overexpression was significantly correlated with larger colorectal tumor size (Abubaker et al. 2009).

##### KRAS signaling and pathway dysregulation

**KRAS activation and upstream regulation** Ras protein is an intracellular membrane-bound guanine nucleotide-binding protein with two states: active (GTP-bound) and inactive (GDP-bound), which it can alternate between. Because of this alternation ability, the Ras protein is called a molecular switch. (Sancho et al. 2004; Simanshu et al. 2017). In the case of *KRAS* inactive state, GTPase-activating proteins (GAPs), such as NF1 and p120-Ras, are responsible for inhibiting the *KRAS*, which is done by the GAPs’ GTPase activity that hydrolyses GTP and catalyzes the γ-phosphate hydrolysis of KRAS bound to GTP, thereby increasing the KRAS GDP-bound ratio. Therefore, no sufficient GTP can bind to the KRAS protein. In contrast, the guanine nucleotide exchange factor (GEF), such as Ras Guanyl nucleotide-releasing protein and Son of Sevenless (SOS) enhances KRAS''''''''s GTP loading efficiency, favoring its active state and promoting the release of GDP to allow GTP binding (Simanshu et al. 2017).

**Fig. 3:**
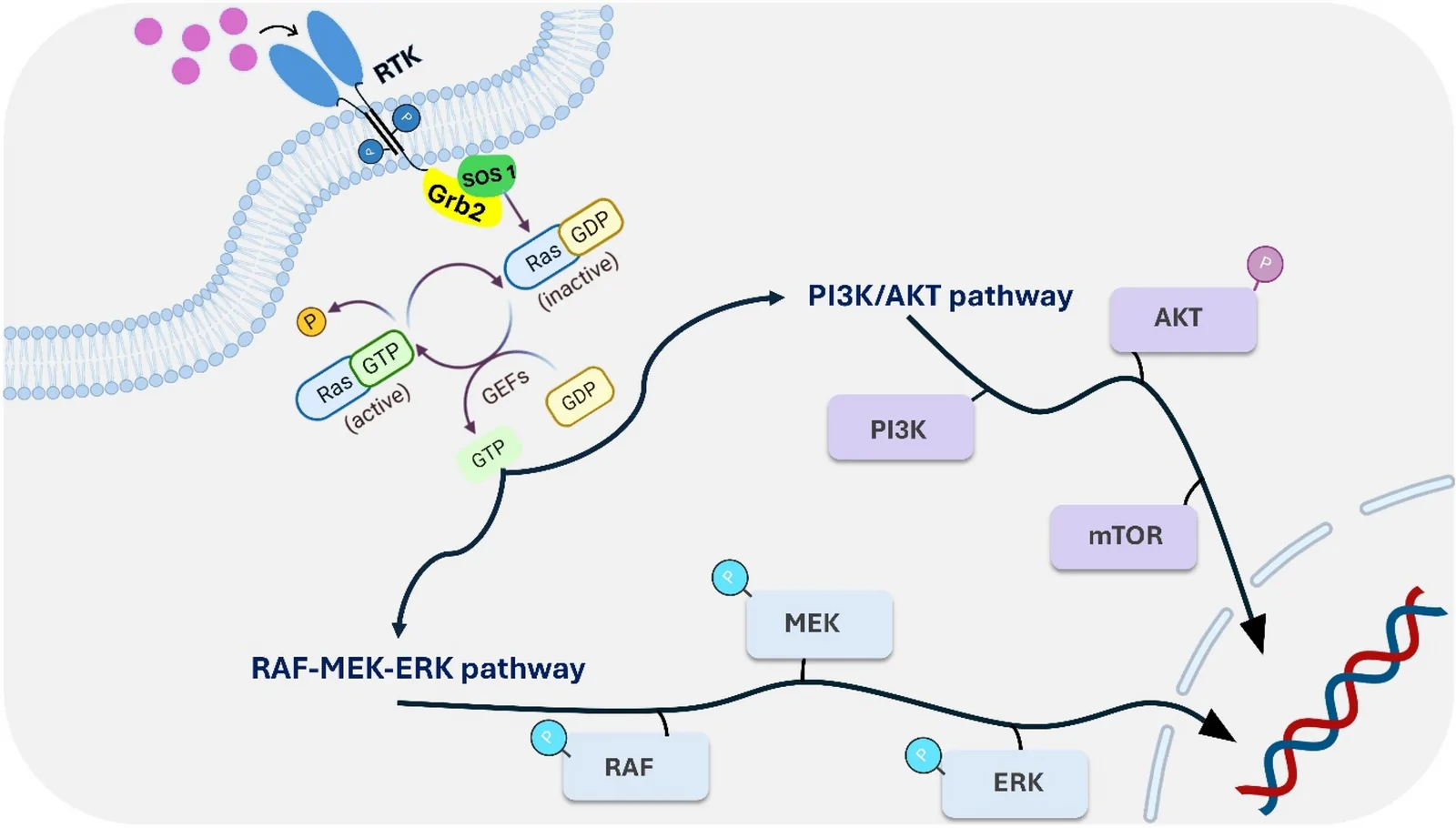
KRAS protein and signal transduction. KRAS is activated downstream of receptor tyrosine kinase (RTK) stimulation, such as EGFR activation, through recruitment of adaptor protein Grb2 and the guanine nucleotide exchange factor SOS1. As a small GTPase, KRAS cycles between an inactive GDP-bound state and an active GTP-bound state, regulated by GEFs and GTPase-activating proteins (GAPs). Activated KRAS engages major downstream signaling pathways, including the RAF–MEK–ERK (MAPK) cascade and the PI3K–AKT–mTOR pathway. These signaling axes regulate cellular processes such as proliferation, survival, cell cycle progression, metabolism, autophagy, apoptosis, migration, and invasion. (Edited with BioRender). Abbreviations: RTK, receptor tyrosine kinase; Grb2, growth factor receptor-bound protein 2; SOS, son of sevenless; GEF, guanine nucleotide exchange factor; GAP, GTPase-activating protein; GTP, guanosine triphosphate; GDP, guanosine diphosphate; RAS, rat sarcoma viral oncogene; RAF, rapidly accelerated fibrosarcoma; MEK, MAPK/ERK kinase; ERK, extracellular signal-regulated kinase; PI3K, phosphoinositide 3-kinase; AKT, protein kinase B; mTOR, mammalian target of rapamycin

On the other hand, the signal transduction cascade of RAS activation starts when an epidermal growth factor (EGF) binds to the epidermal growth factor receptor (EGFR), a receptor tyrosine kinases (RTKs) in the extracellular domain (Fig. 3). The binding of the ligands to their RTK receptors leads to RTK receptor dimerization, which induces conformational changes through autophosphorylation of the receptor intracellular carboxyl-terminal domain. The phosphorylation stimulates docking adaptor proteins having SH2 domains, such as growth factor receptor-bound protein 2 (Grb2), which binds directly to RTKs. Because of this binding, guanine nucleotide exchange factors (GEFs), like SOS, are recruited via RTKs to promote activation of KRAS protein by facilitating GDP-GTP exchange. Thus, downstream pathways such as the RAS–RAF–MAPK pathway activate nuclear transcription factors involved in cell growth, survival, and metastasis. KRAS GTP-bound triggers several downstream pathways (Ferreira et al. 2022; Malumbres and Barbacid 2003; Kim et al. 2021; Pantsar 2020).

**RAF–MEK–ERK signaling pathway** The RAF-MEK-ERK pathway is the first identified Ras pathway effector. When the Ras protein binds to GTP, it attaches to rapidly accelerated fibrosarcoma (RAF), a serine/threonine kinase. This binding relocates RAF to the plasma membrane, which triggers a pathway cascade of the mitogen-activated protein Kinase (MAPK), crucial for the phosphorylation and activation of the MAPK/ERK kinase (MEK). This is followed by extracellular signal-regulated kinase (ERK) phosphorylation and activation via MEK (Malumbres and Barbacid 2003). ERK regulates several downstream processes, including the regulation of many transcription factors and cellular processes like proliferation, cell cycle progression, apoptosis, and autophagy (Ferreira et al. 2022; Parker and Mattos 2018; Lu et al. 2016; Hall et al. 2002).

**PI3K–AKT signaling pathway** Another major Ras pathway effector is the PI3K/AKT pathway. A key product of PI3K activation is phosphatidylinositol-3,4,5-triphosphate (PIP3), whose synthesis results from the phosphorylation of phosphatidylinositol-4,5-biphosphate (PIP2) following the interaction of active KRAS with phosphatidylinositol 3-kinase (PI3K). PIP3 recruits and activates many important proteins, such as mammalian target of rapamycin (mTOR), AKT, and phosphatidylinositol-dependent kinase 1 (PDK1), which have a pleckstrin homology domain to carry out signals that regulate many cellular processes, like proliferation, cell cycle progression, glucose metabolism, autophagy, apoptosis, migration, and invasion (Parker and Mattos 2018; Arrington et al. 2012; Castellano and Downward 2011).

**Oncogenic KRAS activation and clinical implications in CRC** The most prevalent mutation of the *KRAS* oncogene is a mutation in G12C at the 12th position (glycine (G) is substituted with cysteine (C)) (Ostrem et al. 2013). This mutation results in the synthesis of a mutant KRAS protein with altered function, that is continuously active due to defective hydrolysis of GTP, consequently leading to persistent activation of downstream signaling pathways, such as the RAF-MEK-ERK pathway and the PI3K/AKT pathway, which subsequently results in malignant cell growth and proliferation (Chu et al. 2018). KRAS-mutant CRCs are linked to poor tumor differentiation, distant metastasis, advanced disease stage, and worse patient survival. Additionally, cancers with *KRAS* mutations are more resistant to chemotherapy and EGFR-inhibitors-targeted therapy, such as panitumumab and cetuximab, which lowers overall survival (OS) in patients with CRC (Simanshu et al. 2017).

#### p53 signaling pathway

The guardian of the genome and the regulatory master of cellular damage response are names given to the p53 protein, which is crucial for genome stability and cell cycle regulation (Robles 2016). Under different circumstances such as mutagenic stress or nutrient deprivation, stress stimuli, changes in tissue homeostasis, or signals inside the cell, P53 is activated to initiate different expression programs to restore and maintain cellular integrity and biological functions by promoting DNA repair, cell cycle arrest, senescence, metabolism, reactive oxygen species (ROS) production, migration of stem cells, and progenitor cells (Allen et al. 2014; Olivos and Mayo 2016). The tumor suppressor gene *TP53* is located on chromosome 17 on the short arm (17p13.1), which encodes the P53 protein that has at least 12 isoforms generated through alternative promoter usage, alternative splicing, or alternative translation initiation (Joerger and Fersht 2010; Hernández Borrero and El-Deiry 2021).

##### p53 activation and regulation

**Fig. 4:**
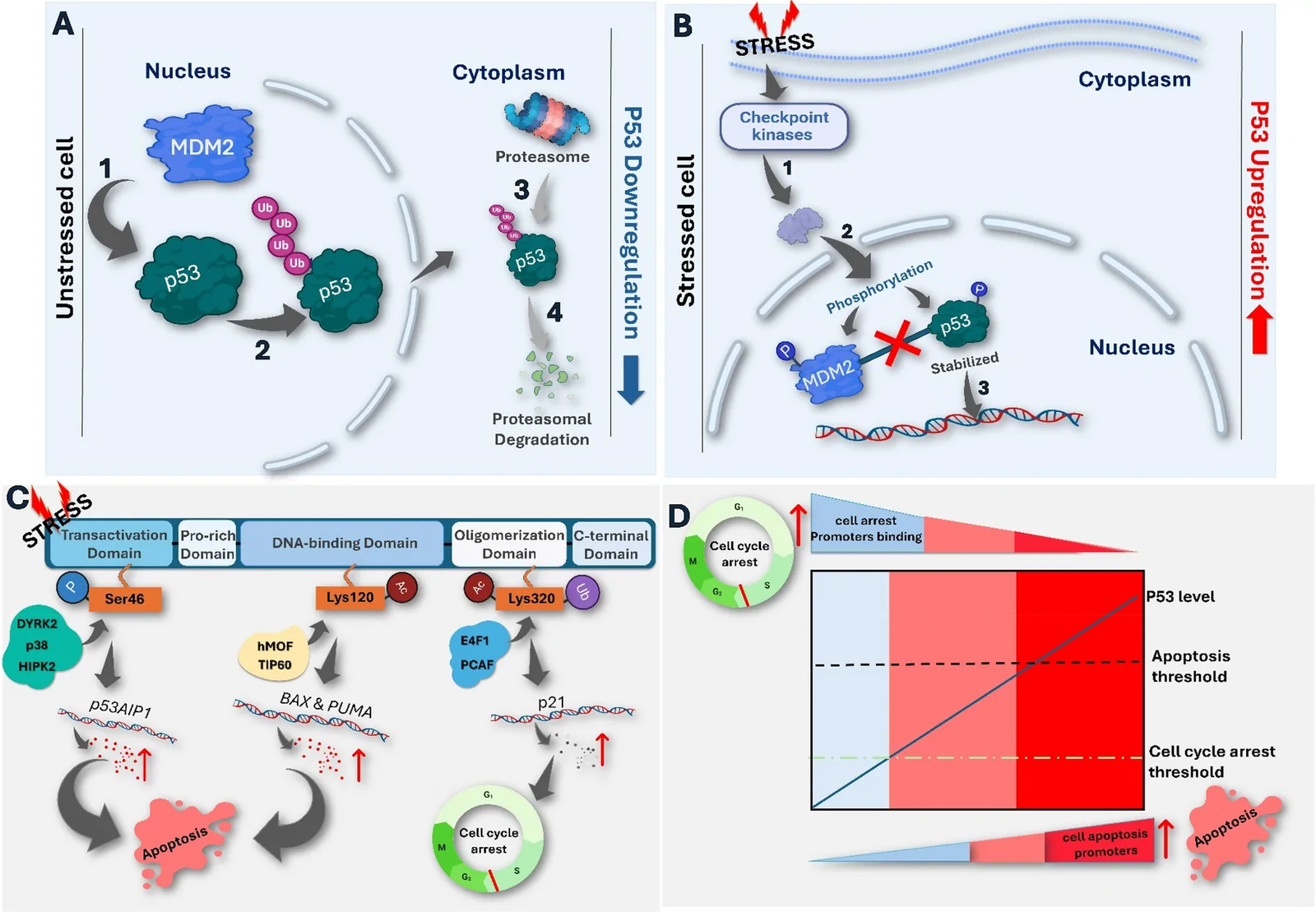
Regulation of p53 signaling and cell fate determination. **A**–**B** MDM2-dependent pathways. A MDM2 binds to p53 and ubiquitinates p53 protein, promoting its nuclear export and proteasomal degradation (negative feedback loop). B Under stress or high DNA damage, the cell’s checkpoint kinases elevate causing phosphorylation of p53 and MDM2, blocking their interaction and causing disruption of their interaction, and p53 stabilizes, which will target and activate genes responsible for apoptosis and cell cycle arrest. **C**–**D** two predictive *p53* gene activation models under cellular stress determine cell fate. C Target gene binding selectivity: Post-translational modifications (PTMs) controlling p53 gene selectivity. In the case of apoptosis, Ser46 phosphorylation at the p53 N-terminal transactivation domain causes selective targeting of the pro-apoptotic gene *p53AIP1*. Whereas DNA binding domain (DBD) Lys120 acetylation activates *BAX* and *PUMA* (pro-apoptotic genes). On the contrary, Lys320 ubiquitination and acetylation in the C-terminal region activate pro-arrest genes (*p21*), which arrest the cell cycle. D The p53-binding site affinity and p53 threshold, under low-stress (low levels of DNA damage) states, p53 protein expression is low and causes cell cycle arrest via binding to high-affinity target genes, allowing cell survival. Long-term DNA damage exposure (higher stress) causes more accumulation of p53 (p53 level elevation), consequently causing its binding to weaker promoters to pro-apoptotic target genes, leading to cell apoptosis. Abbreviations: p53, tumor protein p53; MDM2, mouse double minute 2 homolog; ATM, ataxia telangiectasia mutated; ATR, ATM and Rad3-related; DYRK2, dual-specificity tyrosine phosphorylation-regulated kinase 2; HIPK2, homeodomain-interacting protein kinase 2; p38, p38 mitogen-activated protein kinase; Ser46, serine 46; Lys120, lysine 120; Lys320, lysine 320; ASPP1, apoptosis-stimulating protein of p53 1; hMOF, histone acetyltransferase males absent on the first; TIP60, Tat-interactive protein 60; PUMA, p53 upregulated modulator of apoptosis; E4F1, E4F transcription factor 1; PCAF, p300/CBP-associated factor; DNA, deoxyribonucleic acid

In healthy unstressed cells, p53 transcription factor (TF) activity is tightly regulated because p53 controls the transcription of several target genes in the nucleus, while in the cytoplasm, p53 induces cell death via interacting with the apoptotic machinery proteins (Murray-Zmijewski et al. 2008). Thus, its levels must be low when there are no *p53* activating signals, which is the responsibility of MDM2 (Esser, Scheffner and Höhfeld 2005; Ljungman 2000). In MDM2-dependent pathways, MDM2 binds to the TAD region of p53, which ubiquitinates p53 to promote its nuclear export and proteasomal degradation (Fig. 4.A) (Murray-Zmijewski et al. 2008; Ljungman 2000). This interaction forms an autoregulatory negative feedback loop, in which p53 transcriptionally activates MDM2, and MDM2 negatively regulates p53 protein (Matheu et al. 2008).

In stressed cells, the intensity of the stress signal and the cell type determine the activation of p53. The p53–MDM2 interaction is dissociated via stress-dependent post-translational modifications (PTMs) by checkpoint kinases, causing phosphorylation of p53 (and MDM2), which block their interaction with each other and subsequently, p53 stabilization, and level elevation (Fig. 4B) (Wade et al. 2013). The p53 regulates diverse cellular processes, such as cell cycle arrest, DNA repair, metabolism, reprogramming of stem cells, apoptosis, ferroptosis, metastasis, and invasion. The p53 regulatory mechanisms underlying these heterogeneous outcomes of cell fate determination (life or death) are not clearly understood (Liebl and Hofmann 2021; Carvajal and Manfredi 2013). Many researchers have proposed two potential mechanistic models of gene activation, which are how p53 determines cell fate: (1) Target gene binding selectivity and (2) the p53-binding site affinity (p53 threshold) (Carvajal and Manfredi 2013; Zhao et al. 2000; Weinberg et al. 2005).

#### The p53 target gene binding selectivity

Post-translational modifications (PTMs) are involved in specific target gene selections by p53. Phosphorylation of Ser46 promotes selection of pro-apoptotic target genes such as *p53AIP1* in response to high DNA damage (Oda et al. 2000). In addition, acetylation at Lys120 escalates p53-dependent apoptosis by activating pro-apoptotic genes such as *PUMA* and *BAX*(Figure.C) (Charvet et al. 2011; Hofmann et al. 2002). In contrast, acetylation and ubiquitination of Lys320 lead to activation of pro-arrest genes such as p21 that induce cell cycle arrest (Le Cam et al. 2006; Tang et al. 2006).

Another proposed mechanism of p53 selection is differential activation of p53 target genes, in which the DNA sequence of the p53 response element (*p53RE*) encodes necessary information for associated gene activation, which determines how effectively p53 can bind and activate transcription. However, this mechanism is not clearly understood and requires an in-depth study (Fischer 2019; Riege et al. 2020; Fischer and Sammons 2024).

##### The p53-binding site affinity and p53 threshold

The DNA binding affinity of p53 with target gene recognition sites varies due to the *p53RE* variants. Therefore, some promoters of genes controlling cell cycle arrest have a high affinity for p53 even if a low level of intracellular p53 accumulation due to DNA damage. On the other hand, other gene promoters that promote cell apoptosis have a low affinity for p53, and their activation involves higher levels of p53, which can result from prolonged DNA damage or severe cellular stress (Fig. 4.D) (Weinberg et al. 2005; Inga et al. 2002). Recently published research on whether p53 is smart (selectively activates its target gene) or dumb (p53-binding site affinity determines cell fate choice), states that the cell determines its fate by the threshold-based mechanism rather than the complex p53RE decoding (2024).

##### p53 mutations in CRC and CIN

TP53 has been directly associated with human colon cancer and has a significant role in the development of CIN (Pino and Chung 2010). The CIN pathway is associated with deletion of the chromosome 17p13.1 segment, which contains the tumor suppressor gene *TP53* and *MAP2K4* (Grady 2004; Hoang et al. 2013). In addition, the deletion of chromosome 1q21.3 region that contains gene *S100A2* is associated with CRC (Hoang et al. 2013; Rand et al. 2008).

Taken together, p53 is crucial for genome stability and cell cycle regulation and has a significant role in the development of CIN. However, the p53 regulatory mechanisms underlying these heterogeneous outcomes of cell fate determination (life or death) are not clearly understood, and the mechanisms of p53 target gene binding selectivity and p53-binding site affinity (p53 threshold) require an in-depth study.

#### Microsatellite instability and DNA mismatch repair deficiency in CRC

Short Tandem Repeats (STRs) “exist in more than 100,000 regions of the human genome and are known as microsatellites (Lee et al. 2016). DNA microsatellite regions consist of 1–6 bp tandemly repeated motifs, which are also referred to as perfect tandem repeat (PTR), exact tandem repeat (ETR), tandem repeat (TR), simple sequence repeats (SSRs), or STRs. They constitute about 1–3% of the human genome and are highly abundant in noncoding regions of DNA (Bagshaw 2017). They are also distributed within genes, including introns, untranslated regions (3′-UTRs and 5′-UTRs), and expressed sequence tags (ESTs) (Li et al. 2004). The genomic distribution of simple sequence repeats (SSRs) has significant effects on DNA replication, DNA mismatch repair (MMR) system, cell cycle, recombination, chromatin organization, and regulation of gene activity (Li et al. 2004; Li et al. 2002).

These repeats are used as genetic markers because they provide evolutionary advantages for rapid adaptation for new environments and suffer higher mutation rates than the rest of the genome (Trifonov 2004). They are classified into four main types; perfect, imperfect, interrupted and compound (composite) repeats (Trifonov 2004; Jarne and Lagoda 1996). Microsatellites have captured researchers'''''''' attention for several reasons, such genetic mapping, the investigation of (MSI) in human diseases, population genetics, genotyping, and paternity testing (O’Donnell and Warren 2002; Schlötterer and Harr 2000).

##### DNA replication fidelity

DNA replication infidelity arises from replication slippage or mutations in polymerase delta (POLD1) or epsilon (POLE). In DNA replication slippage, DNA polymerase pauses and dissociates from DNA, particularly at repetitive microsatellite sequences. In replication slippage, the nascent DNA strand temporarily dissociates from the template strand and is rapidly followed by misaligned reassociation, leading to base-pairing errors and the nascent strand loops produce repeat expansions (insertions). On the other hand, with loop occurrence on the template strand, the number of repeat sequences will decrease (deletions) (Hile and Eckert 2004; Oliveira et al. 2006).

Microsatellite stability and frequent C-base to A-base transversions are associated with deficiency in polymerase proofreading activity. Approximately 6% of CRCs harbor somatic *POLE* mutations, whereas a smaller percentage of CRC patients carry *POLD1* mutations (Barbari and Shcherbakova 2017). Because MSI and mismatch-repair testing add cost and turnaround to the diagnostic pathway, there is growing interest in deep learning models that predict MSI, mismatch-repair deficiency and even POLE-mutant status directly from routine hematoxylin and eosin slides, serving as a low-cost pre-screen that concentrates confirmatory molecular testing on the tumors most likely to be positive (see section “Computational and machine learning foundations of CRC molecular classification”).

##### MMR system and MSI development

Several repair mechanisms include MMR, nucleotide excision repair (NER), base excision repair (BER), and interstrand crosslink repair. The MMR is crucial for the cellular post-replication process that maintains DNA replication homeostasis and genome stability through correction of replication slippage and DNA mismatches, including frameshift mutations (insertions/deletions) and base substitutions (Fig. 4 A-B) (Mouw et al. 2017; Liu et al. 2017). In humans, the pathway of MMR contains various proteins, which form heterodimers with diversified functionality combinations according to dimer formation. (Jiricny 2013)

The MSH2 is the main corrective protein, which forms two distinctive heterodimers, the MSH2-MSH3 heterodimer through binding to the MSH3 protein and the MSH2-MSH6 heterodimer through binding to the MSH6 protein, that are respectively called MutSβ and MutSα, whose role is binding to mismatches and checking the post-replicated DNA strand. The MutSα heterodimer detects single-base mismatches and small insertion–deletion loops. whereas the MutSβ heterodimer recognizes larger insertion–deletion loops (up to approximately 13 bases). The MSH5 protein is coupled exclusively with MSH4 and is abundant specifically in mammalian reproductive tissues, and it primarily functions at gene conversions, and crossing-over events.

In humans, several homologs of MutL exist. They are classified among the proteins family gyrase II/Hsp90/histidine kinase/MutL (GHKL) and also function by forming heterodimers, which are MLH1-PMS2 (MutLα) and MLH1-MLH3 (MutLγ) (Jiricny 2013).

**Fig. 5:**
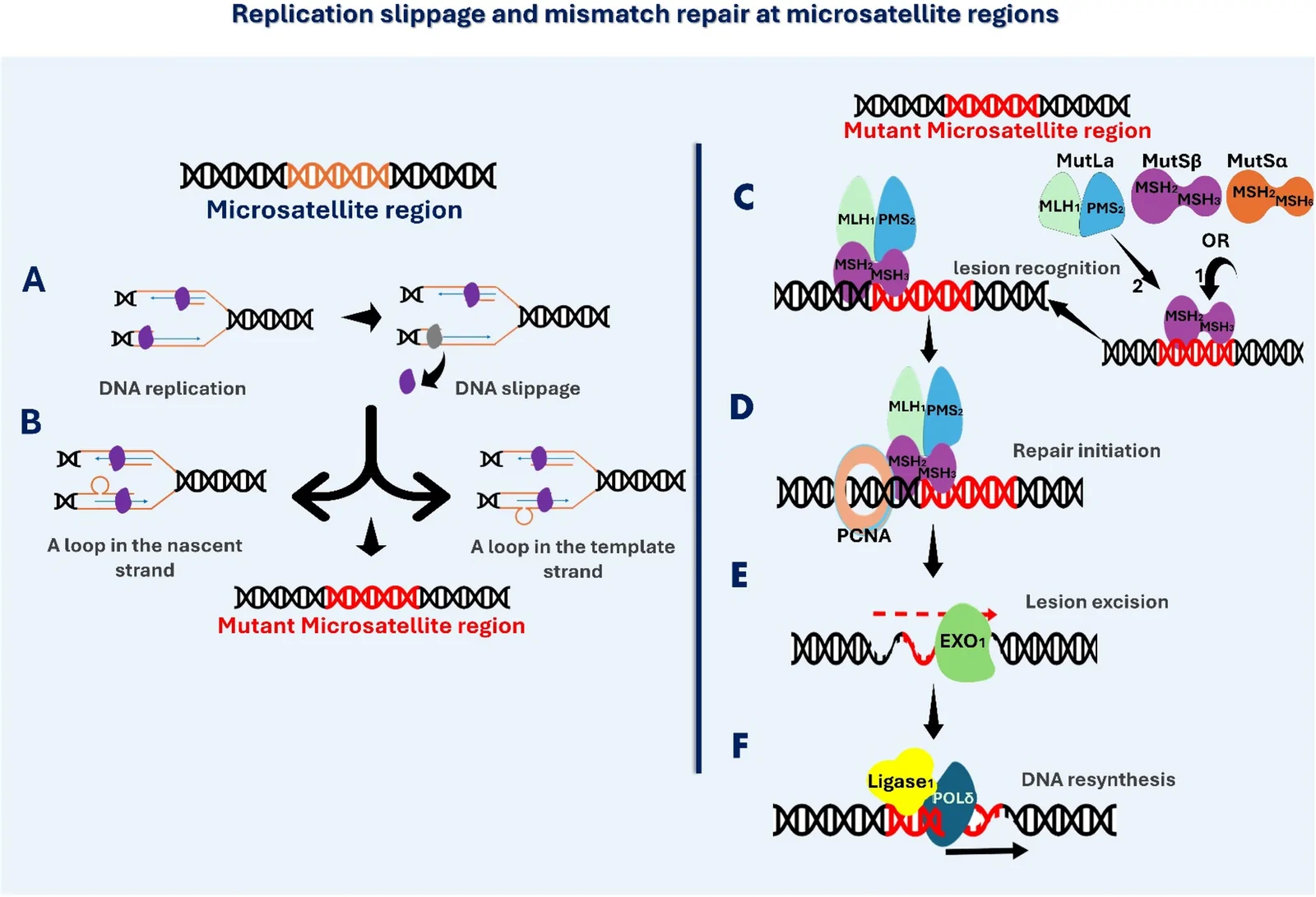
Replication slippage and mismatch repair at microsatellite regions. **A**–**B** Replication slippage. A At microsatellite regions, DNA polymerase may dissociate from DNA. B The loop formation in the nascent strand, will result in repeat extension. Whereas repeats in the template strand will be contracted (decreased) in case of loop formation. This results in microsatellite mutation. **C**–**F** DNA MMR pathway. C MutSa/MutSβ recognizes DNA replication mismatches and binds to it, then recruits MutLα. D MutLα is activated via PCNA to form a single-strand break. E The excision is catalyzed on the nascent strand by the exonuclease EXO1 till shortly after the mismatch. F DNA polymerase δ (POLδ), resynthesis of DNA strand, and Ligase 1 seals DNA nicks. Abbreviations: DNA, deoxyribonucleic acid; EXO1, exonuclease 1; MMR, mismatch repair; MutLα, MutL homolog alpha complex; MutSα, MutS homolog alpha complex; MutSβ, MutS homolog beta complex; PCNA, proliferating cell nuclear antigen; Pol δ, DNA polymerase delta

The proteins of the MMR pathway among all organisms are almost highly conserved and involve four steps: lesion recognition, repair initiation, lesion excision, and DNA resynthesis (Fig. 5C-F) (Pećina-Šlaus et al. 2020; Eso et al. 2020). MMR is crucial for maintaining the microsatellites'''''''' sequence lengths because any defect in this repair mechanism will consequently fail to correct mismatch errors, which the repeat lengths will vary and lead to genomic instability like MSI, and develope tumor cells (Eso et al. 2020; Schmidt and Pearson 2016).

##### Crossing over

Unequal crossing-over can occur due to the presence of microsatellite repetitive regions, which can misalign during synapsis, resulting in chromosomes of unequal length. One of the homologous chromosomes will receive a larger fragment (with a high number of microsatellite repeats), and the other will receive a smaller fragment (with fewer repeats). This change, involving loss or gain of repeats during recombination, occurs through unequal crossover and gene conversion, resulting in microsatellite sequence variation (Oliveira et al. 2006; Majewski and Ott 2000; Ellegren 2004). However, microsatellite mutations arising from recombination errors are less frequent (Ellegren 2004; Huang et al. 2002).

##### MSI in sporadic and hereditary CRC

The two types of CRC on the basis of the MSI pathway are (1) sporadic CRC that has no family history, which is found in approximately 15–20% of CRC cases, due to epigenetic modification through hMLH1 promoter methylation, that causes gene expression inactivation rather than gene mutation and (2) Lynch syndrome, which is a disorder originating from MMR genes germ line mutation (hMLH1, PMS2, MSH6, and hMSH2) (Yamamoto and Imai 2019; Nikanjam et al. 2020). Furthermore, this syndrome is inherited among families and accounts for 3–5% of the approximated CRC patients (Worthley and Leggett 2010;Nikanjam et al. 2020;Lynch et al. 2015;Umar et al. 2004).

According to the MSI frequency, it has three categories: high microsatellite instability (MSI-H), low microsatellite instability (MSI-L), and MSS. MSI-L and MSS are often grouped in current clinical research (Bonneville et al. 2020). MSI-H CRC patients exhibit distinct clinicopathological characteristics compared to those with MSS. MSI-H cancer is located mainly in the colon’s right side with strong lymphocyte infiltration, generally higher than in MSS tumors, although MSS is heterogeneous. Additionally, after surgical intervention, the survival rate of MSI-H carcinoma patients is higher (Buckowitz et al. 2005). It should be noted that immune infiltration is not exclusive to MSI-H tumors, as some MSS subtypes, particularly CMS4 tumors, also exhibit a highly active TME associated with stromal activation, epithelial–mesenchymal transition (EMT), TNF-α/NF-κB signaling, and immune-related signatures, indicating a rich stromal and immune microenvironment (Kantha et al. 2025; Høye et al. 2025). Overall, MMR is crucial for maintaining the microsatellites'''''''' sequence lengths and genome stability, and any defect in this repair mechanism can lead to MSI and genomic instability. However, MSI-H CRC patients exhibit distinct clinicopathological characteristics compared to those with MSS, although MSS is heterogeneous and immune infiltration is not exclusive to MSI-H tumors.

#### CpG island methylator phenotype in CRC

One of the crucial regulatory epigenetic mechanisms of gene expression that induces gene repression is DNA methylation. The CpG islands are regions enriched in CG dinucleotides, which are frequently located in promoter regions and represent hotspots for DNA methylation that induce gene silencing (gene repression) and are found in about 60% of promoters in human genes. The process starts at the CpG site when the DNA methyltransferases (DNMTs) use a methyl donor S-adenosylmethionine (SAM) to add a group of methyl (CH₃) to cytosine’s 5*''''''''* carbon in order of 5-methylcytosine (5mC) formation (Bouyahya et al. 2022). Hypermethylated promoters can be found in 20–30% of CRCs, which occur in genes responsible for cell cycle regulation, apoptosis, and angiogenesis and this hypermethylation causes inappropriate silencing of gene expression (Goel and Boland 2012; Hughes et al. 2012). DNA methylation in CRC can be classified into two types based on tumor progression: (1) Type A (age-related) methylation, in which colon tumor formation is preceded by the methylation predisposition of genes *ESR1, IGF2,* and *TUSC3* (Mojarad et al. 2013; Toyota and Issa 1999; Issa 2000). (2) Type C (cancer-specific) methylation, characterized by the CIMP, in which cytosines in gene promoters become extensively methylated and frequently overlap with MSI-H and a subset of MSS cancers (Taieb et al. 2022).

##### Different CIMP classifications of CRCs

CIMP results in tumor suppressor gene promoter methylation and DNA repair gene methylation, which causes gene silencing (Bouyahya et al. 2022). CIMP is claimed to be associated with older age, tumor location, *BRAF* mutations, MSI, female sex, and, in some cases, *KRAS* mutation (Ushijima and Suzuki 2019). Fang et al. demonstrated that mutant *BRAF*^*V600E*^ activates the transcriptional repressor MAFG, which promotes promoter methylation of CIMP-associated genes, including *MLH1*, leading to *MLH1* silencing and contributing to the development of the CIMP phenotype in CRC. The mutant *BRAF*^*V600E*^ activates the BRAF/MEK/ERK signaling pathway to phosphorylate MAFG, thereby promoting phosphorylation, activation, and increased levels of MAFG, which binds to promoters of CIMP-associated genes, such as *MLH1*. Subsequently, hypermethylation and transcriptional silencing occur after recruitment of a corepressor complex, involving DNMT3B (DNA methyltransferase), CHD8 (chromatin remodeling factor), and BACH1 (MAFG heterodimeric partner) (Fang et al. 2014). Mutant *BRAF*^*V600E*^ is a hallmark of the serrated pathway and has been reported in approximately 90% of CRC cases arising from sessile serrated adenoma (SSA) lesions. The serrated pathway accounts for approximately 15–30% of CRC cases, and is associated with increased cancer-specific mortality (Colussi et al. 2013; Ijspeert et al. 2015; Palma et al. 2019). However, the precise definition of CIMP varies among studies. Therefore, different classifications have been defined for the CIMP. CIMP can be classified as CIMP-positive (CIMP +) and CIMP-negative (CIMP −), based on promoter methylation status rather than solely on *BRAF* mutation (Weisenberger et al. 2006). Shen et al. categorized CIMP1, CIMP2, and CIMP-negative as the three CRC CIMP groups, in which tumors with *BRAF* mutations and MSI fall into the CIMP1 group. While tumors having *KRAS* mutations with a rare occurrence of *TP53* and *BRAF* mutations and low MSI frequency are categorized under the CIMP2 group. Lastly, tumors with high mutation rates in *Tp53* and low mutation rates in BRAF and KRAS mutations, together with MSI, are associated with the CIMP-negative phenotype (Shen et al. 2007). Ogino et al*.* have classified CIMP based on quantitative methylation of fivegene promoters (*NEUROG1, MLH1, CRABP1, CACNA1G*, and *CDKN2A* (p16)) to be categorized as CIMP-high, which have 4 to 5 methylated genes, CIMP-low, which has fewer than 3 methylated genes, and CIMP-0 has no methylated promoters (Ogino et al. 2006). But a year later, Ogino et al*.* added 3 more gene markers that were associated with CIMP classification (*IGF2, RUNX3*, and *SOCS1*), thus CIMP-high has at least 6 or more markers from the eight common promoter methylation markers, CIMP-low has no more than five markers of promoter methylation markers, and CIMP-0 has no methylated promoters (Ogino et al. 2007). In comparison, another study classified the three CIMP groups using genes like *MINT1, MINT2, MINT31*, *p16,* and *hMLH1 (*Barault et al. 2008). Jass classified CRC subtypes based on MSI and CIMP status into (1) MSI-H, CIMP-high, chromosomally stable, with *BRAF* mutation and *MLH1* gene methylation, which are identified as sporadic MSI-H and found in serrated polyps. (2) MSS/MSI-L, CIMP-high, chromosomally stable, with *BRAF* mutation, and *MLH1* partial gene methylation, origin in serrated polyps. (3) MSS/MSI-L, CIMP-low, chromosomal instability, mutation in *KRAS*, and methylation of the *MGMT* gene, which originates in serrated polyps or adenomas. (4) MSS, CIMP-negative, chromosomal instability, which may be MUTYH-associated polyposis, FAP-associated, or sporadic, and originating in adenomas. (5) CIMP-negative, chromosomally stable, MSI-H, with *BRAF* mutation negative, HNPCC, origin in adenomas, also called familial MSI-H CRC (Jass 2007). There are other classifications varying in their methylation markers to be prognostic factors in CRC, Table 3 shows the common methylated markers that have a role in developing CRC.

**Table 3:** Commonly methylated and silenced genes in CRC

**Gene**	**Gene alias**	**Gene location**	**Protein**	**Function**			**Loss of function**
*APC*	GS; DP2; DP3; BTPS2; DESMD; DP2.5; PPP1R46	5q22.2	Adenomatous polyposis coli protein	Antagonize Wnt signaling pathway			Increased Wnt/β-catenin signaling
*MLH1*	CC2; COCA2; HNPCC; MLH-1; hMLH1; HNPCC2; LYNCH2; MMRCS1	3p22.2	DNA mismatch repair protein Mlh1	DNA mismatch repair			Microsatellite instability
*MGMT*	NA	10q26.3	Methylated-DNA–protein-cysteine methyltransferase	Repair of alkylation DNA damage			Increased G > A mutation frequency
*RASSF1*	123F2; RDA32; NORE2A; RASSF1A; REH3P21	3p21.31	Ras association domain-containing protein 1	Potential tumor suppressor. Required for death receptor-dependent apoptosis			Increased RAS/RAF/MAP kinase signaling, death-receptor-dependent apoptosis
*SLC5A8*	AIT; SMCT; SMCT1	12q23.1-q23.2	Sodium-coupled monocarboxylate transporter 1	Tumor suppressor, of colony formation in colon cancer, prostate cancer, and glioma cell lines			increase the colony-forming ability of colon cells
*RUNX3*	AML2; CBFA3; PEBP2aC	1p36.11	Runt-related transcription factor 3	Transcription factor, involved in the control of cellular proliferation and differentiation			Decreased TGF-β/BMP signaling
*SFRP1*	FRP; FRP1; FrzA; FRP-1; SARP2	8p11.21	Secreted frizzled-related protein 1	Modulators of Wnt signaling through direct interaction with Wnts (inhibitor)			Increased Wnt/β-catenin signaling
*CDH1*	UVO; CDHE; ECAD; LCAM; Arc-1; BCDS1; CD324	16q22.1	Cadherin-1	Involved in mechanisms regulating cell–cell adhesions, mobility, and proliferation of epithelial cells			Loss of cell adhesion, possible increased Wnt/β-catenin signaling
*CDH13*	CDHH; P105	16q23.3	Cadherin 13	Calcium-dependent cell adhesion proteins play a role in connecting cells			Increased PI3K/Akt/mTOR signaling, MAPK signaling
*CRABP1*	RBP5; CRABP; CRABPI; CRABP-I	15q25.1	Cellular retinoic acid-binding protein 1	Regulate the access of retinoic acid to the nuclear retinoic acid receptors, promote apoptosis			Not known
*CDKN2A/p16*	ARF; MLM; P14; P16; P19; CAI2; CMM2; INK4; MTS1; TP16; CDK4I; CDKN2; INK4A; MTS-1; P14ARF; P19ARF; P16INK4; P16INK4A; P16-INK4A	9p21.3	Cyclin-dependent kinase inhibitor 2A	Negative regulator of the proliferation of normal cells by interacting strongly with CDK4 and CDK6			Increased cell proliferation
*HLTF*	ZBU1; HLTF1; RNF80; HIP116; SNF2L3; HIP116A; SMARCA3	3q24	Helicase-like transcription factor	Has both helicase and E3 ubiquitin ligase activities, required for transcriptional activation or repression of specific target promoters			Impaired DNA repair
*Gene*	Gene alias	Gene location	Protein	Function			Loss of function
*TIMP3*	SFD; K222; K222TA2; HSMRK222	22q12.3	Metalloproteinase inhibitor 3	Inhibition of MMPs and ADAMs			Increased EGFR signaling, TNF signaling
*ESR1*	ER; ESR; Era; ESRA; ESTRR; NR3A1	6q25.1-q25.2	Estrogen receptor 1	ligand-dependent nuclear transactivation is involved in the regulation of gene expression, proliferation, and differentiation			Loss of estrogen receptor signaling
*CXCL12*	IRH; PBSF; SDF1; TLSF; TPAR1; SCYB12	10q11.21	`Stromal cell-derived factor 1	Activates the C-X-C chemokine receptor CXCR4 to induce a rapid and transient rise in intracellular calcium ions and chemotaxis levels			Increased tumor cell metastases
*IRF8*	CSBP; IRF-8; ICSBP1; IMD32A; IMD32B; H-ICSBP	16q24.1	Interferon regulatory factor 8	Transcription factor			Interferon signaling
*THBS1/TSP1*	TSP; THBS; TSP1; TSP-1; THBS-1	15q14	Thrombospondin-1	Adhesive glycoprotein that mediates cell-to-cell and cell-to-matrix interactions			Decreased TGF-β1 signaling
*DAPK*	DAPK; ROCO3	9q21.33	Death-associated protein kinase 1	involved in multiple cellular signaling pathways that trigger cell survival, apoptosis, and autophagy			Interferon-gamma signaling, TNF alpha signaling, Fas/APO1 signaling
*VIM*	NA	10p13	Vmentin	responsible for maintaining cell shape and integrity of the cytoplasm, and stabilizing cytoskeletal interactions			No known biological effect
*SEPTIN9*	MSF; MSF1; NAPB; SEPT9; SINT1; PNUTL4; SeptD1; AF17q25	17q25.3	Septin-9	involved in cytokinesis and cell cycle control			Impaired cytokinesis and loss of cell cycle control
*CACNA1G*	NBR13; SCA42; Cav3.1; SCA42ND; Ca(V)T.1	17q21.33	Voltage-dependent T-type calcium	Involved in a variety of calcium-dependent processes, including gene expression, cell motility, cell division and cell death			Modulating calcium signaling, which potentially affects cell proliferation and apoptosis
*APBA1*	X11; X11A; LIN10; MINT1; D9S411E; X11ALPHA	9q21.12	Amyloid-beta A4 precursor protein-binding family A member 1	NA			NA
*APBA2*	X11L; MINT2; LIN-10; HsT16821; X11-BETA; D15S1518E; MGC:14,091	15q13.1	Amyloid-beta A4 precursor protein-binding family A member 2	NA			NA
*SOCS1*	JAB; CIS1; SSI1; TIP3; CISH1; SSI-1; TIP-3; AISIMD; SOCS-1	16p13.13	Suppressor of cytokine signaling 1	triggered cell growth enhancement, anchorage-independent growth, and resistance to death stimuli			reduce many oncogenic features and linked to better overall patient survival
*IGF2*	GRDF; SRS3; IGF-II; PP9974; C11orf43	11p15.5	Insulin-like growth factor 2	Has growth-promoting activity, which promotes cellular growth and development			Reduce tumor proliferation, growth and increase patients'''''''' survivability
*NEUROG1*	AKA; ngn1; CCDDRD; Math4C; bHLHa6; NEUROD3	5q31.1	Neurogenin-1	A transcriptional factor that binds to E-box elements			Associated with neoplastic development, from normal mucosa to primary carcinoma, and metastatic colorectal carcinoma

Information presented in this table was compiled from Ref. ( Lao and Grady 2011) and supplemented with annotations from the UniProt Knowledgebase (UniProtKB) and the NCBI Gene database (Bateman et al. 2024; Sayers et al. 2022)

All in all, CIN, MSI associated with DNA mismatch repair deficiency, defects in DNA replication fidelity, and the CIMP represent the major molecular pathways driving CRC development. These alterations in the genomic and epigenetic landscape are implicated in colorectal tumor initiation and progression and contribute to the heterogeneous outcomes of CRC. The different molecular alterations and classifications demonstrate the complexity and heterogeneity of CRC, while their effects on the surrounding TME indicate that these molecular pathways cannot be considered independently from the tumor microenvironment. Consequently, understanding the interactions between tumor cells and the TME has become essential for improving CRC classification, prognostic stratification, and the development of effective therapeutic strategies. Accordingly, in the following section we will discusses the TME, highlighting some of the key cellular and molecular components and their role in CRC development, progression and therapy response.

### Tumor microenvironment in CRC

Recently, CRC progression and metastasis have no longer been viewed as an epithelial-driven mechanism but as communication between the cancer cells and the surrounding normal stroma within the TME, which supports tumor cell survival, proliferation, and metastatic progression. In CRC, the TME is composed of a heterogeneous network of cellular components like fibroblasts, immune cells, endothelial cells, and extracellular matrix (ECM), as well as gut microbiota also plays a role in tumor growth and progression (Li et al. 2022). Deviations in tumor cells’ transcriptional programs are influenced by epigenomic dysregulation, which affects the TME and its cellular components, ultimately shaping tumor heterogeneity and adaptive responses; therefore, epigenetic therapy has become involved in cancer treatment (Yang et al. 2023). As reviewed previously, recent studies have further refined our understanding of the TME by identifying novel regulatory axes and immune evasion mechanisms (Yang et al. 2023; Chen et al. 2021; Talaat et al. 2022). Among these regulatory mechanisms, aberrant DNA methylation has emerged as a key modulator of the TME.

Aberrant methylation is one of the key regulators modulating TME via recruitment of immune cell by regulating production of cytokines and antigen presentation. For instance, hypermethylation of epidermal growth factor receptor (EGFR) and hypomethylation of hepatocyte growth factor receptor (c-MET) are highly correlated with stage, decreased OS among CRC patients, characterized by immune infiltration, aberrant T-cell phenotypes, and adverse prognostic outcomes (Lawal et al. 2021).

Beyond these methylation-associated alterations, metabolic regulation also contributes to epigenetic remodeling within the TME. DNA methylation can be influenced by nutrient availability, particularly methionine metabolism, which generates S-adenosylmethionine (SAM) as a methyl donor for DNMT1 activity. This increases the methylation of sirtuin 6 (SIRT6) and suppresses its expression, consequently increasing proprotein convertase subtilisin/kexin type 9 (PCSK9) expression, which plays a major role in cholesterol homeostasis. PCSK9 reduces the recycling of the low-density lipoprotein receptor (LDLR) to the cell membrane, thereby reducing cellular cholesterol uptake. In tumor cells, PCSK9 is aberrantly elevated, which aids in tumor progression and inhibits apoptosis in multiple tumors, including CRC, while contributing to cholesterol deficiency in tumor-infiltrating CD8⁺ T cells. Consequently, this compromises T-cell receptor signaling pathways and induces a state of exhaustion, thus weakening antitumor immunity. Beyond this, PCSK9 facilitates immune evasion through two primary avenues: first, it binds to MHC I to trigger its lysosomal localization and degradation, diminishing tumor antigen presentation. Second, it interferes with anti-tumor immunity by inhibiting LDLR-mediated TCR recycling, which compromises CD8⁺ T-cell signaling. These immunosuppressive mechanisms contribute to T-cell exhaustion and immune evasion within the TME, highlighting the importance of immunotherapeutic strategies aimed at restoring effective anti-tumor T-cell responses. In this context, immune checkpoint blockade therapy has transformed cancer treatment by enhancing the ability of the immune system to recognize and eliminate tumor cells (Wang et al. 2025; Azmi et al. 2025).

One of the important pathways is the programmed cell death protein 1 (PD-1) and programmed death ligand 1 (PD-L1). In many cancers, PD-L1 is overexpressed on tumor cells and interacts with PD-1 on activated T cells, resulting in suppression of T-cell activity and immune escape. Immune checkpoint inhibitors are monoclonal antibodies designed to interrupt this PD-1/PD-L1 interaction, thereby reactivating cytotoxic T-cell–mediated anti-tumor immunity (Abaza et al. 2023).

Building upon these immune regulatory mechanisms, these findings suggest a promising new way to treat "cold" MSS CRC tumors that usually show poor response to immunotherapy. Both dietary methionine restriction (DMR) and PCSK9 inhibition prime MSS CRC to respond to PD-1 blockade. Additionally, a triple-threat approach, combining PCSK9 inhibition, PD-1 blockade, and 5-fluorouracil (5-FU) chemotherapy, effectively stunts tumor progression in MSS CRC cases (Wang et al. 2025).

Metabolic remodeling in CRC also occurs under hypoxic conditions, which promote lactate production through aerobic glycolysis. Lactate subsequently activates GPR81, leading to extracellular vesicle production, enhanced tumor malignancy, and chemotherapy resistance through regulation of the MMR system. These metabolic adaptations contribute to CRC progression and tumor adaptation (Li et al. 2025; Aldeyarbi et al. 2026).

In addition to host metabolic pathways, environmental factors within the TME, particularly the gut microbiota, significantly shapes the epigenetic landscape of CRC. Dysbiosis within the microbiome is closely linked to aberrant DNA methylation flux in the colonic mucosa, contributing to histological transformation and malignancy. Compared to healthy controls, CRC-associated microbes induce increased gene hypermethylation, specifically compromising tumor suppressor pathways. Several CRC-associated bacterial species have been implicated in these epigenetic alterations. For instance, colonic mucosa was found to suffer alterations in gene methylation/demethylation and DNA damage because of CRC–associated microbiota (CRC-μ), such as *Bacteroides fragilis* and *Parvimonas micra*, whose abundance is correlated with highly methylated CRC (Bray et al. 2024). For example, multiple promoters were found to be hypermethylated, including WIF1, SEPT9, *ALX4, NPY, PENK, and SFRP*1, 2, and 3 in CRC-μ (Sobhani et al. 2019). In another study, the hypermethylation activity of *P. micra* promotes PI3K-Akt signaling cytoskeleton rearrangement, tumor progression, and invasion (Bergsten et al. 2023). Consistent with these findings,, other bacteria have been linked to hypermethylation of tumor suppressor gene (TSG) promoters in CRC tissue, such as *Hungatella hathewayi*, *Fusobacterium nucleatum*, and *Parvimonas* species. In vitro studies have shown that *F. nucleatum* promotes DNA methyltransferase activity. Further analysis revealed that *Streptococcus* spp. and *H. hathewayi* are significantly associated with hypermethylation of *MLH*1 and *CDX2*, respectively. Altogether, these results show that microbiota-driven epigenetic changes do more than just promote cancer cell growth; they also help remodel the tumor microenvironment. Essentially, the microbiota plays a dual role in both driving tumor progression and altering its local microenvironment (Xia et al. 2020). One notable example is *F. nucleatum* has been shown to promote a pro-inflammatory TME in ApcMin/+ mouse models by recruiting tumor-infiltrating immune cells, generating a pro-inflammatory microenvironment that supports colorectal neoplasia progression (Kostic et al. 2013). Beyond direct immune-cell recruitment, microbial metabolites also contribute to TME remodeling. Moreover, tumor immune microenvironment (TIME) is greatly influenced by microbial metabolite succinate secretion produced by *F. nucleatum* and *H. hathewayi*. Succinate produced by *F. nucleatum* downregulates the expression of *CCL5* and *CXCL10* as a result of attenuation of the cGAS-IFN-β signaling pathway. Consequently, this reduces the infiltration of CD8⁺ T-cells, resulting in weakening of anti-PD-1 therapy efficacy (Jiang et al. 2023). A similar metabolite-driven mechanism has also been reported that succinate produced by *H. hathewayi* binds to the succinate receptor SUCNR1 (also called GPR91) on intestinal epithelial and CRC cells, thereby priming the NLRP3/ASC inflammasome. This leads to caspase-1 activation and maturation/secretion of pro-inflammatory cytokines IL-1β and IL-18, contributing to a pro-tumorigenic inflammatory microenvironment (Zhang et al. 2026).

Collectively, these results show that epigenetic and microbiota-driven alterations do more than just promote CRC progression; they also help remodel the TME. Aberrant DNA methylation, metabolic regulation, and microbial metabolites influence immune infiltration, T-cell activity, antigen presentation, and inflammatory pathways, thereby contributing to immune evasion and adverse prognostic outcomes. These findings further support the importance of therapeutic strategies aimed at restoring effective anti-tumor immune responses within the tumor microenvironment.

### From biomarkers to therapy: actionable targets in CRC

The molecular alterations discussed throughout this review have direct implications for clinical decision-making and therapeutic intervention in CRC. Several molecular biomarkers, including MSI, *KRAS, NRAS, BRAF, HER2*, CIMP, and CMS classification, are associated with disease progression, prognosis assessment, therapeutic response, and patient stratification (Crutcher et al. 2022; Zhang et al. 2025). In addition, these biomarkers provide important information regarding the molecular characteristics and biological heterogeneity of CRC, which can be utilized for selecting appropriate therapeutic strategies. This shift from molecular biomarkers to clinical targets has been revolutionizing the therapeutic arena for CRC, allowing clinicians to better understand which treatments may be suitable for different patients. Progress in genomics, epigenetics, and immuno-oncology has led to an understanding of the major factors controlling tumor biology and responses to treatments. Despite these advances, surgical resection remains the main curative and most effective treatment for early-stage CRC and locally advanced cancers (Mao et al. 2025).

For advanced CRC treatment, several drugs have been approved by the US Food and Drug Administration (FDA), including trifluridine/tipiracil hydrochloride (TAS-102), oxaliplatin, irinotecan, fluorouracil (FU), and capecitabine. These agents are commonly administered as combination chemotherapy regimens, such as FOLFOX (folinic acid, fluorouracil, and oxaliplatin) and FOLFIRI (folinic acid, fluorouracil, and irinotecan). Furthermore, several biologic agents, including ziv-aflibercept, regorafenib, ramucirumab, panitumumab, cetuximab, and bevacizumab, have also been approved. Moreover, first-line treatment of advanced CRC using chemotherapy combined with biologic agents has shown significant improvements in OS (Bennouna et al. 2013; Mahipal and Grothey 2016).

In metastatic colorectal cancer (mCRC), the first-line treatment regimen is selected according to the tumor''''''''s molecular profile and primary tumor location. Most patients receive combination chemotherapy with either an anti-vascular endothelial growth factor (VEGF) antibody or, in patients with RAS wild-type left-sided tumors, an anti-epidermal growth factor receptor (EGFR) antibody. Patients with MSI-H/dMMR tumors are preferentially treated with immunotherapy (immune checkpoint inhibitors). Notably, most patients eventually develop acquired resistance and experience disease progression, typically within 8–14 months of initiating first-line therapy (Morris et al. 2023). In addition, metastatic colorectal cancer (mCRC) continues to be associated with high mortality rates, and finding a treatment with durable and long-term responses remains challenging.

In patients with *RAS* wild-type tumors lacking *PIK3CA, BRAF, NRAS,* and *KRAS* gene mutations, anti–epidermal growth factor receptor (EGFR) monoclonal antibodies, such as panitumumab and cetuximab, have shown effectiveness in this group of patients. These agents act by blocking the RAS/RAF/MAPK/ERK signaling pathway by binding to the EGFR ligand-binding domain and inhibiting receptor autophosphorylation, subsequently inducing receptor endocytosis and degradation, thereby inhibiting tumor cell proliferation and survival. Therefore, anti-EGFR antibodies are combined with standard chemotherapy for primary CRC and mCRC (Abbas et al. 2026). Also, left-sided tumors have demonstrated higher responsiveness than right-sided tumors (Von Einem et al. 2014). However, the toxicity profile of anti-EGFR agents includes stomatitis, diarrhea, infusion-related reactions, and dermatologic reactions (rash, acneiform dermatitis), which may impact patient quality of life (Mahipal and Grothey 2016). In addition, tumor cells commonly demonstrate intrinsic resistance due to insufficient receptor depletion after the treatment course (ALTamimi et al. 2026; Okada et al. 2017). However, in the presence of mutation biomarkers *HER2*, *PIK3CA*, *BRAF*, *NRAS*, and *KRAS*, patients cannot be treated with anti-EGFR antibodies due to upregulation in KRAS signaling, which leads to continuous activation of the RAS/RAF/MAPK/ERK signaling pathway even when EGFR receptors are blocked; subsequently, the tumor bypasses blocked EGFR receptors, driving relentless tumor growth (Afrǎsânie et al. 2019). Thereby, the approved therapy in the first-line setting is the combination of chemotherapy with bevacizumab, the efficacy of which has been demonstrated by multiple retrospective analyses of randomized trials (Mahipal and Grothey 2016).

Tumors harboring the *BRAF *^*V600E*^ mutation are linked to MSI, CIMP, older age, female sex, high-grade mucinous histology, right-sided tumors, and mCRC. Anti-VEGF monoclonal antibody (bevacizumab) has been shown to be the best targeted therapy for this patient group, and studies such as BRiTE and ARIES suggest that patients with *RAS* mutations may also benefit from VEGF inhibitors, particularly when continued beyond progression in combination with chemotherapy (Tsai et al. 2019). Bevacizumab works by targeting the tumor microenvironment, specifically by blocking angiogenesis through vascular endothelial growth factor A (VEGF-A) inhibition, which prevents VEGF-A from interacting with VEGFR on endothelial cells; therefore, it blocks endothelial cell proliferation and new blood vessel formation, leading to slowed tumor growth and reduced metastatic potential (Naz et al. 2024).

In two phase III clinical trials, the combination of EGFR-targeted agents with bevacizumab-plus chemotherapy has been investigated. There was no improvement in survival outcomes, and in some cases, it was associated with a detrimental effect. In the CAIRO-2 trial, patients were randomized to receive XELOX plus bevacizumab with or without cetuximab; no significant differences were observed between the two groups. However, a hazard ratio of 1.15 indicated a survival advantage for the group receiving chemotherapy with bevacizumab alone (Tol et al. 2009). Similarly, the addition of panitumumab to FOLFOX plus bevacizumab and FOLFIRI plus bevacizumab in the PACCE (Panitumumab Advanced Colorectal Cancer Evaluation) trial demonstrated worse survival outcomes, with hazard ratios of 1.89 and 1.28, respectively (Hecht et al. 2009).

However, in patients harboring MSI-H or mismatch repair-deficient (dMMR), such as PMS2, MLH1, MSH2, and MSH6 gene mutations, clinical benefits sustained from immune checkpoint blockade treatments have been demonstrated. Immune checkpoint inhibitors work by blocking inhibitory signaling pathways that normally suppress T-cell activation, thereby restoring cytotoxic T-cell–mediated anti-tumor immune responses (Kuske et al. 2022). In MSI-H/dMMR mCRC patients who have progressed after chemotherapy with irinotecan, oxaliplatin, and fluoropyrimidines, the FDA has approved treatment with immune checkpoint inhibitors, including pembrolizumab and nivolumab, either as monotherapy or in combination with low-dose ipilimumab. The reason behind the sensitivity of MSI-H/dMMR tumors to immunotherapy is due to the accumulation of insertion–deletion mutations, particularly within microsatellite regions that result from deficiencies in the DNA mismatch repair system. Subsequently, this results in the creation of new abnormal proteins (neoantigens), which are then presented by molecules of tumor cell human leukocyte antigen (HLA) and can be recognized by cytotoxic T cells. Consequently, these tumors are characterized by a “hot” TME because of active immune response with CD8⁺ cytotoxic T cell infiltration, but the tumor cells start a feedback response to this immune pressure; thus, inhibitory checkpoint molecules such as cytotoxic T lymphocyte‐associated antigen‐4 (CTLA-4), PD-1, and PD-L1 are upregulated to suppress T-cell activity in the TME (Zhang et al. 2017). To enhance anti-tumor immune responses and enable prolonged disease control via increasing activation and T‐cell proliferation, therapeutic checkpoint inhibitors are used for this patient group (Morse et al. 2020). Some side effects arise within 12 weeks after starting treatment with checkpoint inhibitors, including adrenal insufficiency, hypophysitis, thyroid disease, pancreatitis, nausea/vomiting, colitis, diarrhea, dry skin, and rash, which are immune-related toxicities. Patients may need continuous hormone replacement therapy, but for the other side effects, most of them resolve within 12 weeks of onset (Morse et al. 2020).

### Potential adjuvant therapies in CRC

Some medications have been repurposed in clinical trials as adjuvant therapies, with modest effects. In a clinical trial (phase II), refractory CRC patients received a combined regimen of intravenous irinotecan (125 mg/m2) with oral metformin (2500 mg) daily, achieving a disease control rate (DCR) of 41%, with a median progression-free survival of 3.3 months and OS of 8.4 monthsPatients with *RAS* mutations showed poorer OS. Grade 3 diarrhea (~ 29%) was the most common adverse effect (Bragagnoli et al. 2021). However, the study lacked a control arm. Another clinical trial (phase II) in 50 patients with mCRC received 5-fluorouracil combined with metformin, demonstrated limited overall benefit, with low DCR (22%) and short median progression-free survival (PFS) (~ 2 months), but patients who achieved early disease stabilization experienced prolonged disease control. Multiple adverse effects have been shown among patients, including myelotoxicity, vomiting, nausea, and diarrhea. The limitation of this study is attributed to tumor molecular heterogeneity and the absence of validated biomarkers to guide patient selection (Miranda et al. 2016).

NSAIDs, such as aspirin and COX-2 inhibitors, have been used in tumor treatment due to their availability and low cost, with anticancer effects, especially in *PIK3CA*-mutated tumors (Blanchard-Cavagis et al. 2026). For instance, in a randomized, placebo-controlled, double-blind trial, patients with *PIK3CA* mutations (exon 9 or 20) and stage II or III colon tumors or stage I, II, or III rectal tumors were assigned in a 1:1 ratio to receive placebo or aspirin (160 mg) daily for 3 years. CRC recurrence was significantly lower in patients who received aspirin than in the placebo group (Martling et al. 2025). Moreover, according to a non-randomized retrospective observational cohort study examining whether aspirin use is associated with improved OS in 1,529 patients with mCRC, particularly those with *PIK3CA* mutations in exon 9 or exon 20, or other PI3K pathway mutations, such as *PTEN* and *PIK3R1*. Aspirin was taken for at least 30 days after diagnosis. Patients with *PIK3CA* and PI3K pathway mutations, specifically patients not receiving aspirin, were associated with poorer survival. Among patients who took aspirin, there was no statistically significant survival difference between those with and without *PIK3CA* mutations. Therefore, the findings are hypothesis-generating rather than practice-changing and support the need for prospective randomized studies to determine whether aspirin benefits patients with PI3K/*PIK3CA*-mutant mCRC (Payapwattanawong et al. 2026). In addition, in a meta-analysis of randomized controlled trials (RCTs), 363 patients received a placebo, and 426 patients were assigned to NSAIDs (aspirin or COX-2 inhibitors) to evaluate the therapy outcome in patients with *PIK3CA*-mutated CRC. Adjuvant NSAID therapy was associated with improved disease-free survival (DFS); however, its benefit on OS remains uncertain (Blanchard-Cavagis et al. 2026). Overall, the effect of NSAID therapy on OS in patients with *PIK3CA*-mutated CRC remains uncertain despite encouraging findings for DFS among certain patients.

Combining stereotactic body radiotherapy (SBRT) with sorafenib in a phase II clinical trial showed reduced resistance in patients with CRC liver metastases to radiotherapy. Due to the dual effect of sorafenib and SBRT in inhibiting SLC7A11 expression, which in turn increases CRC cell sensitivity via induction of ferroptosis, the combination was effective. Clinically, the combination overcame resistance to radiotherapy and showed favorable efficacy with a manageable safety profile (He et al. 2025). However, the current evidence remains limited, and larger prospective studies are required to determine whether ferroptosis-based therapeutic approaches can achieve durable clinical benefits across biologically heterogeneous CRC populations.

Putting all these studies together, the therapeutic advances illustrate the growing importance of molecular stratification in CRC management. However, treatment responses remain strongly influenced by tumor heterogeneity, dynamic evolutionary processes, and the emergence of resistance mechanisms.

### Emerging therapeutic strategies and precision oncology in CRC

**Fig. 6:**
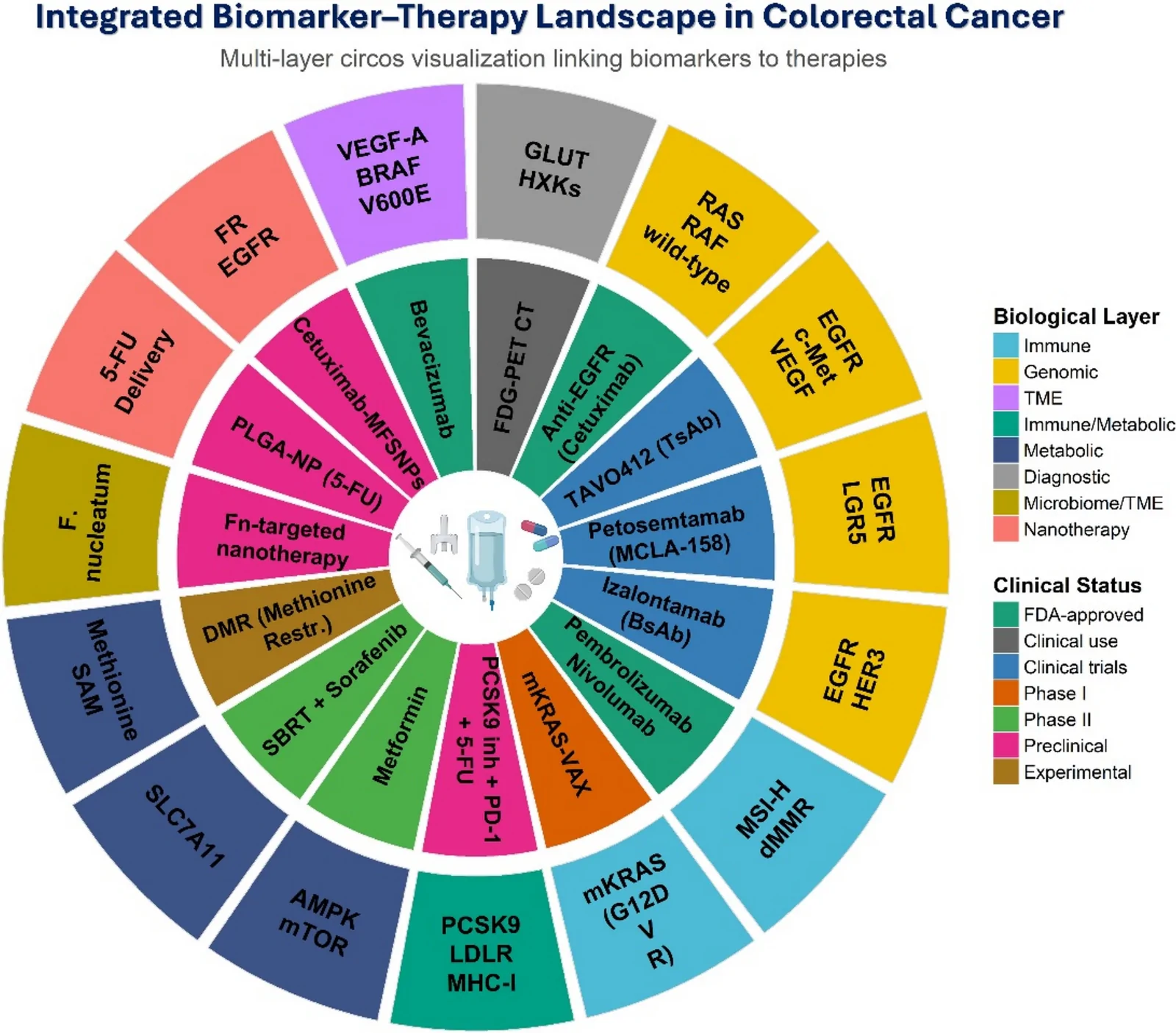
Biomarker-guided therapeutic landscape in colorectal. This figure presents a structured overview of clinically relevant and emerging therapeutic strategies in CRC, mapped to their corresponding predictive biomarkers and biological layers, as discussed in this manuscript. Abbreviations: AI, artificial intelligence; BRAF, v-Raf murine sarcoma viral oncogene homolog B1; CIN, chromosomal instability; CIMP, CpG island methylator phenotype; CRC, colorectal cancer; ctDNA, circulating tumor DNA; dMMR, deficient mismatch repair; EGFR, epidermal growth factor receptor; FDA, U.S. Food and Drug Administration; HER2, human epidermal growth factor receptor 2; KRAS, Kirsten rat sarcoma viral oncogene; MSI-H, high microsatellite instability; NTRK, neurotrophic tyrosine receptor kinase; PD-1, programmed cell death protein 1; PD-L1, programmed death-ligand 1; PI3K, phosphoinositide 3-kinase; POLE, DNA polymerase epsilon; RTK, receptor tyrosine kinase; TME, tumor microenvironment; VEGF, vascular endothelial growth factor

New developments in CRC (CRC) studies have led to a shift in emphasis from traditional treatment modalities to more mechanism-based personalized medicine techniques. Such personalized medicine incorporates immunotherapy and biologic targeting modalities as well as advancements in diagnosis to counter drug resistance and improve efficacy (Fig. 6).

#### Personalized neoantigen and peptide-based cancer vaccines

Researchers are prioritizing peptide vaccines for targeted cancer therapy. Peptide-based therapies eradicate CRC by reprogramming the TME via stimulating the cancer-immunity cycle with tumor-specific antigen presentation. Personalized peptide vaccines (PPVs) have emerged as a potent therapeutic strategy for MSI-H CRC. On the other hand, MSS CRC displays poor sensitivity to immune checkpoint blockade, which underlines the importance of exploring novel approaches such as personalized neoantigen-based vaccination. This clinical difference is partly associated with the lower immunogenicity and reduced neoantigen burden of MSS tumors compared to MSI-H CRC. Tumor-specific antigen (TSA)-based vaccines have transitioned into clinical practice and show measurable efficacy in the management of CRC. Personalized neoantigen vaccines have been applied in MSS-advanced CRC patients. Two-thirds of patients (66.67%) developed a neoantigen-specific immune response following vaccination. This immune activation correlated with better outcomes: responders achieved a median PFS of 19 months, compared to just 11 months for non-responders (Yu et al. 2023). Moreover, mutant *KRAS* (m*KRAS)* neoantigen vaccines targeting (mKRAS-VAX) have been used in the NCT 04117087 trial (phase 1). This synthetic long peptide (SLP) vaccine targets a pool of six prevalent *KRAS* mutations (G12A, G12C, G12D, G13D, G12V and G12R) and is administered in conjunction with dual checkpoint blockade (ipilimumab and nivolumab). The m*KRAS* vaccine demonstrated manageable toxicity and strong immunogenicity, with 8 out of 12 patients showing significant T-cell activation, which 75% of participants exhibited a KRAS-specific T-cell response. Clinical outcomes included a 41.7% DCR and a 24.9-month median OS, with a primary safety profile of Grade 1–2 adverse events (Wang et al. 2025). According to preclinical or clinical research, personalized neoantigen vaccines provided a dual advantage over conventional vaccines by enhancing safety against autoimmune side effects and overcoming immune tolerance (Huang et al. 2025). Interestingly, some studies have investigated autophagy modulation as a novel strategy to enhance tumor antigenicity (Mokarram et al. 2017; Mahgoub et al. 2022).

Overall, personalized peptide and neoantigen vaccines demonstrated strong immunogenicity with manageable toxicity by inducing tumor-specific immune responses. However, PPVs have mainly shown efficacy in MSI-H CRC, whereas personalized neoantigen vaccines have been explored to overcome the poor immunogenicity of MSS CRC, and mKRAS-VAX specifically targets recurrent *KRAS* mutations in combination with immune checkpoint blockade. Despite these promising findings, their efficacy remains dependent on neoantigen immunogenicity and patient-specific immune responses.

#### Next-generation antibody therapeutics

Following the development of personalized neoantigen vaccines, next-generation antibody therapeutics have emerged as another precision immunotherapy strategy to overcome the resistance of anti-EGFR monoclonal antibodies drugs (panitumumab and Cetuximab) in mutate *PI3K*, *NRAS*, and *KRAS* patients, bispecific/trispecific antibodies (BsAbs/TsAbs) and antibody–drug conjugates (ADCs) have been introduced in clinical trials. For instance, TAVO412 is the first trispecific antibody (TsAb) to target EGFR, c-Met, and VEGF simultaneously, offering a triple-action approach that shuts down key growth signals (EGFR and c-Met signaling) and prevents the formation of new tumor blood vessels (Shang et al. 2026).

Izalontamab (SI-B001) is an EGFR/Her3 BsAbs, which has the ability to target both EGFR/HER3 heterodimers and EGFR/EGFR homodimers by blocking them with an anti-EGFR IgG1 antibody and an anti-Her3-scFv fragment fused to the antibody C-terminus. A study of 60 patients showed the drug is safe as a monotherapy, with no reported fatalities despite common side effects like rash and paronychia. Among the 16 patients who achieved stable disease, one was a CRC patient. These initial findings justify evaluating the drug’s potential in combinatorial regimens (Xue et al. 2025).

The antibody drug, Petosemtamab (MCLA-158), has been evaluated in human CRC organoid models and is characterized by enhancing antibody-dependent cellular cytotoxicity. In experimental settings, Petosemtamab has shown significant tumor-suppressing capabilities, regardless of whether a KRAS mutation was present or not. Different from cetuximab or a parental isotype drug, Petosemtamab promotes the internalization and degradation of EGFR. The efficacy of the drug in treating CRCs has also been observed in xenograft and organoid models, where it has been found to be superior to other treatments (Slobodnyuk et al. 2025). In the NCT03526835 phase II clinical trial, the efficacy and safety of petosemtamab (1500 mg Q2W) were evaluated as monotherapy or in combination with chemotherapy (FOLFOX/FOLFIRI) in BRAF-, NRAS-, and KRAS-wild-type patients with left- or right-sided MSS metastatic CRC. Across multiple lines of therapy, petosemtamab was effective and well-tolerated. When combined with chemotherapy in first- and second-line settings, patients achieved promising response rates. No drug-related mortality was observed; however, some adverse effects arose from the drug combination (all grades [G]/G3), including peripheral neuropathy (43%/0%), fatigue (43%/0%), constipation (43%/0%), and dermatitis acneiform (71%/0%) (Khushman et al. 2025).

Overall, next-generation antibody therapeutics demonstrated enhanced antitumor activity with manageable safety by overcoming the limitations of conventional anti-EGFR therapies. However, BsAbs/TsAbs simultaneously target multiple signaling pathways, whereas Petosemtamab promotes EGFR internalization and degradation, providing distinct mechanisms to suppress tumor growth. Despite these promising findings, current evidence is mainly limited to preclinical and early-phase clinical studies. Future studies should validate these approaches in larger clinical trials and optimize combination strategies, particularly for patients harboring *PIK3CA*, *NRAS*, and *KRAS* alterations.

#### Theranostics and molecular imaging-guided therapy

Building on the development of next-generation antibody therapeutics, theranostics and molecular imaging-guided therapy further advance precision medicine by integrating targeted diagnosis with personalized treatment. This approach relay on integrating therapy and non-invasive diagnostic approaches into a single platform, as in theranostic nanoparticle therapy, is a necessity to drive the biomedical industry toward a new era of personalized medicine (Kasi et al. 2023). Multiple imaging techniques can be used to detect tumor tissue localization and visualize the TME, such as Positron Emission Tomography (PET), Single Photon Emission Computed Tomography (SPECT), Magnetic Resonance Imaging (MRI), Computed Tomography (CT), and near-infrared (NIR) fluorescence imaging. These molecular imaging techniques rely on tumor-specific biomarkers, which are important for achieving high diagnostic accuracy and therapeutic efficacy. In targeted therapy, these imaging modalities are combined with therapeutic strategies, including chemotherapy, nanomedicine, radiotherapy, photothermal therapy (PTT), and photodynamic therapy (PDT), in which they rely on theranostic agents that serve as both diagnostic and therapeutic agents (Menon et al. 2025). Consequently, molecular imaging and theranostic nanomedicine have emerged as promising strategies for improving patient stratification, treatment monitoring, and personalized management of CRC.

For instance, in clinical diagnosis, the combination of noninvasive imaging techniques, such as PET for imaging tumor metabolic activity, and CT for detailed anatomy, to detect tumor activity, metastases, and recurrent tumors, has changed treatment decisions in about 1/3 of advanced rectal cancer patients (Heriot et al. 2004). One of the PET radiotracers that is widely used in cancer detection is Fluorodeoxyglucose (FDG). Cancer cells have a high demand for glucose; thus, they overexpress glucose transporters (GLUTs) and enzymes such as hexokinase (HXKs). When FDG, a glucose analog in which one hydroxyl group is replaced by radioactive fluorine-18, is injected, cancer cells take it up more aggressively than normal tissue. However, FDG becomes trapped inside the cells, after being phosphorylated to FDG-6-phosphate by HXKs and cannot continue through metabolic pathways. Thereby, this leads to the accumulation of FDG in cancer cells compared to surrounding tissues, and the tumor then appears as “hot spots” on the resulting images (Izuishi et al. 2014).

PET-CT is used as a quantitative measurement of tumor viability and metabolic activity by measuring ^18^F-FDG accumulation via the maximum standardized uptake value (SUVmax). However, Natural glucose activity in the gut, especially near the colon and small intestine, can cause background noise on PET/CT scans, potentially leading to inaccurate diagnoses (Heriot et al. 2004). Moreover, a retrospective study on 164 CRC patients concluded that PET-CT is less effective for early-stage disease and should be restricted to high-risk populations and yields higher prognostic outcomes when combined with tumor markers such as carcinoembryonic antigen (CEA) and carbohydrate antigen 19–9 (CA19-9) (Fujimoto et al. 2025). Nevertheless, FDG-based PET/CT remains valuable for evaluating tumor metabolic activity, monitoring therapeutic response, and assessing advanced or metastatic CRC. To improve CRC detection, a promising biomarker has been developed, 18F-RWY, a PET radiotracer that specifically targets the integrin α6 protein. Because this protein is a marker for aggressive mCRC, its effectiveness has only been evaluated in mouse models (Xiao et al. 2019). For better CRC management, in a study, researchers recommend the use of two tracers: 68Ga-FAPI for imaging cancer-associated fibroblasts (CAFs) in TME, and FDG for evaluating tumor metabolic behavior in PET/CT. Nevertheless, mCRC patients showed poor prognosis and suppressive tumor immunity with higher 68Ga-FAPI uptake. However, a combination of immunotherapy using TGF-βR inhibitor with KN046 (targeting both PD-L1 and CTLA-4) and 68Ga-FAPI PET/CT imaging assists in monitoring tumor response, and CAF levels change during therapy, making it a valuable method for guiding and adjusting CRC immune treatments (Li et al. 2024).

A lot of studies are involved in designing nanoparticles that can serve as theranostics, to serve both for diagnostics and therapeutics. In addition to conventional targeted antibody therapies, EGFR-targeting strategies have also been integrated into theranostic nanoparticle platforms for simultaneous imaging and treatment of CRC. One of the methods developed is targeting overexpressed receptors in CRC compared to normal cells, such as the Folate receptor alpha and EGFR, which makes these receptors attractive targets for selective imaging and drug delivery with minimal toxicity to healthy tissues. For instance, magneto-fluorescent nanoparticles (MFSNPs), combined with cetuximab (Ctx), an anti-EGFR antibody, have been developed to target overexpressed Folate receptor (FR) and EGFR receptors in CRC, leading to enhanced accumulation at the tumor site and producing strong MRI signals for effective imaging. However, it has only been tested in preclinical mouse models of CRC (Cho et al. 2010).

Furthermore, research is focusing on using nanoparticle-based delivery systems. One of the polymers that has been approved by the FDA is poly(D,L-lactide-co-glycolide) (PLGA). PLGA has been used in nanodrug delivery for its biodegradability, biocompatibility, and efficient protection of the loaded anticancer drug. In CRC targeted therapy, PLGA-based NPs have been used to encapsulate 5-FU to boost the anticancer effectiveness of the chemotherapy. A hydrophilic polar polymer, such as Polyethylene glycol (PEG), is conjugated to PLGA to improve nanoparticle stability, circulation time in the blood, and minimize immunogenicity (El-Hammadi et al. 2017).

Targeting ligands are required to deliver nanoparticles to the tumor site, as they enhance nanoparticle recognition and internalization. These ligands can include polysaccharides, transferrin, antibodies, integrins, or folic acid. Folic acid (FOL) is widely incorporated into nanoparticles to target colon cancer cells because folate receptors (FRs) are highly expressed on the surface of cancer cells and are expressed at low levels in normal cells. In addition, FOL is a natural ligand, a water-soluble vitamin B, which makes it suitable for use in drug delivery systems to direct nanoparticles to cancer cells that overexpress folate receptors. Binding to folate receptors on cancer cells increases the uptake of 5-FU by tumor cells and enhances cell growth inhibition (El-Hammadi et al. 2017; Handali et al. 2018).

Another type of nanocarrier commonly used in targeted drug delivery is liposomes. They have several advantages, including the ability to encapsulate both hydrophilic and lipophilic drugs, low toxicity, biodegradability, and biocompatibility. However, loading hydrophilic drugs into liposomes remains challenging during formulation. This is because such drugs are highly water-soluble and tend to remain dissolved in the surrounding aqueous phase during liposome preparation rather than being efficiently encapsulated (Handali et al. 2018; Yang et al. 2015). Additionally, liposomes may undergo uptake by circulating myeloid cells of the mononuclear phagocyte system, which can influence biodistribution, reduce targeting efficiency, and potentially contribute to off-target side effects (Huang et al. 2019).

Taken together, molecular imaging and theranostic nanomedicine have emerged as promising strategies for improving patient stratification, treatment monitoring, and personalized management of CRC. However, different imaging modalities and theranostic platforms rely on distinct tumor-specific biomarkers and therapeutic approaches to achieve targeted diagnosis and treatment. Despite these promising findings, many theranostic agents and nanoparticle platforms have only been evaluated in preclinical models, while others remain limited by diagnostic sensitivity and drug delivery efficiency. Therefore, future studies should focus on validating these approaches in clinical trials and optimizing biomarker-guided theranostic platforms to improve the personalized management of CRC.

#### Microbiome-targeted therapeutic

Microbiome-targeted therapeutics in CRC have recently emerged as a promising precision medicine strategy. The gut microbiota is associated with the CRC immune microenvironment, carcinogenesis, immune modulation, and metabolic reprogramming, which shape tumor progression and therapeutic responses. This approach aims to overcome pathogen-driven immunosuppression and chemoresistance by selectively eliminating oncogenic bacteria while simultaneously restoring antitumor immune responses.

*F. nucleatum* is one of the primary pathogens implicated in CRC immunosuppression and chemoresistance, posing a formidable challenge to therapeutic efficacy. Therefore, researchers have focused on developing a single nanocarrier containing both antitumor agents and antibacterial drugs, which can target bacterial aggregation and tumor cells to eradicate them. The specificity and tumor selectivity of the nanodrug are due to its acidity-activated charge-conversion mechanism, which targets the acidic environment found in tumor tissue. When the nanodrug reaches the targeted site, it eradicates both bacteria and CRC cells, triggering the immune system through the release of DAMPs (damage-associated molecular patterns) resulting from cancer cell death, and PAMPs (pathogen-associated molecular patterns) resulting from *F. nucleatum* death. Subsequently, this promotes the activation and maturation of dendritic cells (DCs) and stimulates T-cell infiltration into the tumor and additionally inhibits epithelial–mesenchymal transition (EMT). Thus, this multifunctional nanopeptide stimulates the host immune response, enhancing immunotherapy and suppressing Fn-colonized mCRC (Wei et al. 2026).

For instance, one of the emerging CRC-targeted therapies is nanotechnology-based microbiota-targeted platforms, which use nanoparticle delivery systems to amplify anti-tumor immunity, remodel the TME, and restore microbial homeostasis (Li et al. 2026). The lactic acid bacterium *Limosilacto-bacillus reuteri* (*L. reuteri*) has been used, in which immunoadjuvant (CpG)- and carbon dot (CD)-loaded plant lipid nanoparticles (CD/CpG@LNPs) are conjugated to the bacterial surface through reactive oxygen species (ROS)-responsive linkers. This lipid- and protein-based nanomaterial regulates microbiota-associated metabolic pathways, induces ROS-mediated immunogenic cell death (ICD), and enhances CD8⁺ T-cell tumor infiltration by releasing neoantigens and promoting CpG-mediated DC maturation. Thus, it suppresses tumor growth and CRC liver metastasis (Li et al. 2026; Xu et al. 2025).

Moreover, in CT26 tumor–bearing mice, regorafenib (REG)-loaded nanoparticles based on an amphiphilic inulin–deoxycholic acid conjugate (UIRN) effectively altered the gut microbiota by increasing beneficial bacteria and short-chain fatty acid (SCFA) production while reducing tumor-promoting microbial populations. Consequently, modulation of the gut microbiota together with REG delivery promoted polarization of tumor-associated macrophages, thereby reducing immunosuppression and enhancing anti-tumor immunity. Intriguingly, polymer-based nanomedicines are superior to lipid- and protein-based systems. Inulin is an FDA-approved drug delivery biopolymer that is used in colon-specific drug release, which protects encapsulated drugs from the acidic and enzymatic conditions of the upper gastrointestinal tract. Microbial degradation of inulin promoted localized release of the nanomedicine within CRC lesions, resulting in more effective inhibition of tumor growth (Zhu et al. 2024).

Another microbiome-targeted therapeutic strategy currently under clinical investigation aims to improve chemotherapy efficacy by modulating gut microbial composition in mCRC. Preclinical studies have demonstrated that increased colonization by *F. nucleatum* and *E. coli* is associated with chemoresistance and unfavorable clinical outcomes. Therefore, an ongoing phase II clinical trial (NCT06728072) is evaluating microbiome modulation therapy (MBMT) in adults with stage IV mCRC receiving first-line 5-FU-based chemotherapy. Patients are randomized to receive standard chemotherapy alone or in combination with MBMT, consisting of ciprofloxacin, metronidazole, with or without aspirin, administered for 28 days. The study will evaluate objective response rate (ORR), OS, PFS, safety, and tolerability, while exploratory analyses will assess alterations in gut microbiota composition and diversity using shotgun metagenomic sequencing. This approach may provide a novel strategy to overcome microbiota-associated chemoresistance and improve therapeutic outcomes in mCRC (Kinsey et al. 2026).

### Clinical challenges and future perspectives

Despite the significant advances in the understanding of CRC molecular pathogenesis and the development of targeted therapeutic approaches, several clinical challenges continue to limit treatment efficacy and patient outcomes. Tumor heterogeneity, therapeutic resistance, and the complex interactions within the TME remain a major challenge in CRC management that limit precision medicine strategies.

For instance, a precise diagnosis of CRC is crucial in all cases. Patients normally undergo mismatch repair protein testing, such as MSI testing or mismatch repair immunohistochemistry (MMR IHC). These tests are important for screening patients for Lynch syndrome (hereditary tumors) and identifying tumors that may benefit from immunotherapy (Openshaw et al. 2023). Approximately 85% of annual CRC cases are MSS, having poor response to immune checkpoint inhibitors (ICIs) in mCRC, while MSI-H/dMMR cases have high response rates to ICIs because they possess high tumor mutational burden (TMB), enhanced immunogenicity, and increased neoantigen load (Saeed et al. 2026; Koustas et al. 2026). Accordingly, no standard immunotherapy strategy for MSS/pMMR has been established. Hence, MSS/pMMR tumors have a low neoantigen load and TMB; tumor cells have limited immunogenicity and are less visible to cytotoxic T cells. Multiple signaling pathways, including PI3K/AKT, MAPK, WNT/β-catenin, and TGF-β, contribute to the major resistance mechanisms in MSS/pMMR tumors. Among these, TGF-β-driven stromal exclusion is considered the predominant mechanism. Increased *TGF-β* expression promotes stromal remodeling through the activation of CAFs, leading to the accumulation of collagen fibers and fibronectin within the extracellular matrix, thereby restricting the infiltration of cytotoxic T cells (Sahin et al. 2025). To overcome the poor native response of MSS tumors to immunotherapy, multiple combinatorial strategies with immune checkpoint inhibitors have been clinically tested to improve tumor immunogenicity, particularly in patients with MSS/pMMR metastatic CRC. For example, in the phase II AtezoTRIBE trial, the addition of atezolizumab to FOLFOXIRI plus bevacizumab demonstrated clinical benefit regardless of the presence of liver metastases (Antoniotti et al. 2025). In another phase I study (NCT03860272), the combination of botensilimab and balstilimab treatment for MSS mCRC was promising (Bengala et al. 2026). However, several drug combination strategies for MSS/pMMR CRC patients are still being evaluated and have not yet demonstrated sufficient evidence to alter standard clinical practice (Koustas et al. 2026).

Real-world evidence highlights several limitations in the current biomarker-guided management of pMMR/non-MSI-H mCRC patients. Using the iKnowMed electronic health record database, which has the records of previously treated pMMR/non-MSI-H mCRC patients and their evaluation for biomarker testing patterns and treatment outcomes. About 72% of cases received specific biomarker testing, and about 70% received common anti-cancer drugs (TAS-102 and regorafenib). While a few cases received supportive care only to relieve symptoms, this accounted for about 29%. About 1.7% didn''''''''t receive treatment. Most patients underwent further biomarker testing, including *KRAS* and *BRAF*. The OS was about 7.4 months on average, while the disease control period was 3.5 months until progression. It has been found that a large percentage of patients did not undergo biomarker testing that could help in determining their treatment. Moreover, many patients did not receive any recommended treatment in later lines of therapy, such as regorafenib or TAS-102, after progression from standard chemotherapy. This suggests that there is an insufficient treatment strategy for these patients (Desai et al. 2025).

Several emerging biomarkers can contribute to patient selection and treatment personalization beyond the existing biomarkers in CRC. TMB and *POLE/POLD1* mutations have been shown to have significant potential in defining MSS/pMMR tumors. However, their use is impeded by inconsistent predictive value and the low frequency of these alterations. While circulating tumor DNA (ctDNA) has already proven its capacity to assess minimal residual disease, response to therapy, and tumor evolution, it should be further investigated for its predictive value in immunotherapy (Ma et al. 2022; Kulangara et al. 2019; Zhou et al. 2025). Moreover, the gut microbiome appears to play a role as an immune modulator in cancer, with ongoing efforts to develop therapies that alter the microbiome components, such as *F. nucleatum* and *E. coli*, to improve the efficacy of immunotherapy, as mentioned in the previous section (Li et al. 2026). Genomic, immune, and microbiome-related biomarkers are expected to allow more precise patient stratification and facilitate personalized treatment design in CRC in the future. Further clinical trials will test different combinations of immunotherapeutic drugs and targeted treatments, as well as new biomarker-guided approaches, especially among patients with MSS/pMMR CRC who lack efficient immunotherapy (Koustas et al. 2026).

Future developments will likely focus on integrating multi-omics data, digital pathology, AI-based approaches, and high-throughput technologies to improve the accuracy, accessibility, and clinical applicability of CRC molecular classification. The major challenge in precision oncology is not only identifying actionable biomarkers but also integrating genomic, epigenetic, and TME information to improve patient selection and develop more durable therapeutic strategies. In addition, expanding access to comprehensive medical records and establishing international data-sharing networks are essential to support large-scale analysis and ensure that advances in molecular classification and personalized medicine can benefit patients worldwide. AI and high-throughput technologies may facilitate large-scale comparisons of treatment regimens and improve the identification of molecular subtypes associated with specific therapeutic responses. However, in mCRC, the complex interactions between genetic alterations, epigenetic changes, and environmental factors that contribute to treatment resistance and relapse remain incompletely understood. Future studies should focus on improving immunotherapy strategies, including the identification of novel biomarkers, optimization of combination therapies, and expanding clinical trials to enhance the benefit of immunotherapy across different CRC subtypes. Furthermore, emerging therapeutic approaches, including radiotherapeutic strategies, may provide additional opportunities to improve outcomes for patients with mCRC.

### Limitations of the study

This review has some limitations, however. For one thing, as a narrative synthesis, it draws on previous literature based on heterogeneous study designs, populations, and technologies used, which could influence the variability of results. Secondly, even though the present review provides information from the standpoint of molecular pathways and TME, most mechanisms mentioned in this review – especially microbiome-related mechanisms and nanoparticle therapy – rely mostly on pre-clinical evidence.

Another limitation refers to how incomplete our understanding of TME features like immune cell plasticity and microbiome dynamics are, which could lead to incomplete modeling of their impact on CRC pathogenesis and treatment options. Moreover, there is an absence of well-established and clinically validated biomarkers of emerging pathways, which could impede their use in precision oncology applications. Finally, though promising approaches to therapy have been highlighted, more research is needed to understand their potential long-term efficacy and resistance mechanisms.

## Conclusion

The CRC pathogenesis is characterized by gradual accumulation of molecular changes, resulting in the deregulation of essential cellular processes, such as proliferation, genome integrity, programmed cell death, and differentiation. Signaling pathways, such as the Wnt/β-catenin pathway, RAS-RAF-MAPK pathway, PI3K/AKT pathway, and DNA mismatch repair, play an important role in the development of the adenoma–carcinoma sequence and CRC pathobiology. With recent advances in the molecular classification of colorectal tumors, additional insights into CRC heterogeneity and the biological differences among tumor subtypes have been established. Particularly, the introduction of the CIMP pathway and its connection to MSI, BRAF mutations, and serrated lesions has added another layer of complexity to the molecular characterization of CRC.

In addition, understanding colorectal carcinogenesis requires not only characterization of molecular alterations but also consideration of their temporal sequence during disease development. Longitudinal molecular epidemiology and prospective cohort-based approaches, including the PCIBM. Moreover, molecular profiling of the tumor and its microenvironment is a necessity today in determining how to best tailor treatments like targeted therapy and immunotherapy, especially for individuals with MSI-H/dMMR, MSS/pMMR as well as those with oncogenic driver mutations. Additionally, there have been advances in precision oncology, such as targeted therapy, immune checkpoint inhibition, peptide vaccines, antibodies of the next generation, molecular imaging, and microbiome-based therapy, that allow personalized therapies for CRC. Continued research integrating molecular profiling, biomarker discovery, and precision medicine approaches will further expand this repertoire of therapeutic strategies and improve clinical outcomes for patients with CRC.

## Data Availability

No datasets were generated or analysed during the current study.

## Abbreviations

AKT: Protein Kinase B
APC: Adenomatous Polyposis Coli
ASPP1: Apoptosis-Stimulating Protein of p53 1
ATM: Ataxia Telangiectasia Mutated
ATR: ATM and Rad3-Related
BAX: BCL2-Associated X Protein
BER: Base Excision Repair
BMP: Bone Morphogenetic Protein
BRAF: V-Raf Murine Sarcoma Viral Oncogene Homolog B1
CAAX: Cysteine–Aliphatic–Aliphatic–Any Amino Acid Motif
CHK1/CHK2: Checkpoint Kinase ½
CIMP: CpG Island Methylator Phenotype
CIMP-H: High CpG Island Methylator Phenotype
CIN: Chromosomal Instability
CK1: Casein Kinase 1
CMS: Consensus Molecular Subtypes
CMS1: Consensus Molecular Subtype 1
CMS2: Consensus Molecular Subtype 2
CMS3: Consensus Molecular Subtype 3
CMS4: Consensus Molecular Subtype 4
CNV: Copy Number Variation
AI: Artificial Intelligence
CRC: Colorectal Cancer
CTD: C-Terminal Domain
ctDNA: Circulating Tumor DNA
DBD: DNA-Binding Domain
dMMR: Deficient Mismatch Repair
DNA: Deoxyribonucleic Acid
DNMT: DNA Methyltransferase
DL: Deep Learning
DYRK2: Dual-Specificity Tyrosine Phosphorylation-Regulated Kinase 2
E4F1: E4F Transcription Factor 1
FDA: U.S. Food and Drug Administration
EGFR: Epidermal Growth Factor Receptor
ERK: Extracellular Signal-Regulated Kinase
EST: Expressed Sequence Tag
EXO1: Exonuclease 1
FAP: Familial Adenomatous Polyposis
GAP: GTPase-Activating Protein
GDP: Guanosine Diphosphate
GEF: Guanine Nucleotide Exchange Factor
Grb2: Growth Factor Receptor-Bound Protein 2
GSK-3: Glycogen Synthase Kinase 3
GTP: Guanosine Triphosphate
HER2: Human Epidermal Growth Factor Receptor 2
HIPK2: Homeodomain-Interacting Protein Kinase 2
hMOF: Histone Acetyltransferase Males Absent on the First
HNPCC: Hereditary Nonpolyposis Colorectal Cancer
HVR: Hypervariable Region
iCMS: Intrinsic Consensus Molecular Subtype
iCMS2: Intrinsic Consensus Molecular Subtype 2
iCMS3: Intrinsic Consensus Molecular Subtype 3
imCMS: Image-based consensus molecular subtype
KRAS: Kirsten Rat Sarcoma Viral Oncogene
LOH: Loss of Heterozygosity
MAPK: Mitogen-Activated Protein Kinase
MEK: MAPK/ERK Kinase
MMR: Mismatch Repair
MSI: Microsatellite Instability
MSI-H: High Microsatellite Instability
MSI-L: Low Microsatellite Instability
MSS: Microsatellite Stable
MutLα: MutL homolog alpha complex
MutSα: MutS homolog alpha complex
MutSβ: MutS homolog beta complex
mTOR: Mammalian Target of Rapamycin
NF1: Neurofibromin 1
NER: Nucleotide Excision Repair
NTRK: Neurotrophic Tyrosine Receptor Kinase
OD: Oligomerization Domain
PCNA: Proliferating Cell Nuclear Antigen
PDK1: Phosphoinositide-Dependent Kinase 1
P38: P38 Mitogen-Activated Protein Kinase
PCAF: P300/CBP-Associated Factor
PDS: Pathway-Derived Subtypes
PD-1: Programmed Cell Death Protein 1
PD-L1: Programmed Death-Ligand 1
PDS1: Pathway-Derived Subtype 1
PDS2: Pathway-Derived Subtype 2
PDS3: Pathway-Derived Subtype 3
PI3K: Phosphoinositide 3-Kinase
PIP2: Phosphatidylinositol-4,5-Bisphosphate
PIP3: Phosphatidylinositol-3,4,5-Triphosphate
POLE: DNA Polymerase Epsilon
POLD1: DNA Polymerase Delta 1
POLδ: DNA Polymerase Delta
PRD: Proline-Rich Domain
PTM: Post-Translational Modification
PUMA: P53 Upregulated Modulator of Apoptosis
RAF: Rapidly Accelerated Fibrosarcoma
RAS: Rat Sarcoma Viral Oncogene
ROS: Reactive Oxygen Species
RTK: Receptor Tyrosine Kinase
SAM: S-Adenosyl Methionine
scRNA-seq: Single-Cell RNA Sequencing
SOS: Son of Sevenless
SSR: Simple Sequence Repeat
STR: Short Tandem Repeat
TAD: Transactivation Domain
TCF/Lef: T-Cell Factor/Lymphoid Enhancer Factor
TCGA: The Cancer Genome Atlas
TGF-β: Transforming Growth Factor Beta
TIP60: Tat-Interactive Protein 60
TME: Tumor Microenvironment
TP53: Tumor Protein p53
UTR: Untranslated Region
VEGF: Vascular Endothelial Growth Factor
Wnt: Wingless/Integrated Signaling Pathway
